# The Role of Microbiota in the Pathogenesis of Bullous Pemphigoid and Pemphigus Vulgaris: Evidence, Controversies, and Perspectives

**DOI:** 10.3390/ijms26136076

**Published:** 2025-06-24

**Authors:** Francesca Gorini, Alessio Coi, Michele Santoro, Alessandro Tonacci, Francesco Sansone, Elena Biancamaria Mariotti, Marta Donati, Alice Verdelli, Maria Rita Nasca, Paolo Amerio, Emiliano Antiga, Emanuela Barletta, Marzia Caproni

**Affiliations:** 1 Unit of Epidemiology of Rare Diseases and Congenital Anomalies, Institute of Clinical Physiology, National Research Council, 56124 Pisa, Italy; alessio.coi@cnr.it (A.C.); michele.santoro@cnr.it (M.S.); 2 Information Systems Unit, Institute of Clinical Physiology, National Research Council, 56124 Pisa, Italy; alessandro.tonacci@cnr.it (A.T.); francesco.sansone@cnr.it (F.S.); 3 Department of Health Sciences, Section of Dermatology, Azienda USL Toscana Centro, University of Florence, 50122 Florence, Italy; elenabiancamaria.mariotti@unifi.it; 4 Department of Health Sciences, Section of Dermatology, University of Florence, P. Palagi Hospital, 50122 Florence, Italy; elettradonati@gmail.com (M.D.); alice.verdelli@hotmail.it (A.V.); emiliano.antiga@unifi.it (E.A.); marzia.caproni@unifi.it (M.C.); 5 Dermatology Clinic, University of Catania, 95123 Catania, Italy; mnasca@tiscali.it; 6 Dermatologic Clinic, G. D’Annunzio University, 66100 Chieti, Italy; p.amerio@unich.it; 7 Department of Experimental and Clinical Biomedical Sciences “Mario Serio”, Experimental Pathology and Oncology Section, University of Florence, 50134 Firenze, Italy; emanuela.barletta@unifi.it

**Keywords:** bullous pemphigoid, pemphigus vulgaris, autoimmune bullous skin diseases, gut microbiota, oral microbiota, skin microbiota, probiotics

## Abstract

Bullous pemphigoid (BP) and pemphigus vulgaris (PV) represent the most prevalent conditions among autoimmune bullous skin diseases, considered a major cause of severe morbidity and, in certain cases, mortality. The hallmark of the two diseases is the presence of autoantibodies directed against proteins located in the basement membrane of the skin, which determines the formation of blisters. In recent years, interest in the role of microbiota in relation to health-disease status has progressively increased. In particular, based on the gut–skin axis, accumulating evidence has emerged on the potential association between the composition and diversity of microbial communities in the gut, skin, and even in the oral cavity and the risk of developing BP and PV. Dysbiosis, characterized by a generally higher relative abundance of Firmicutes and a depletion of probiotics/beneficial species, might contribute to the pathogenesis of both diseases. Despite the still limited number of studies and the need for further large-scale multicenter studies, the knowledge gathered so far is suggestive of a novel modifiable risk factor representing a potential target for adjuvant treatments of these disabling and life-threatening conditions.

## 1. Introduction

Autoimmune bullous skin diseases, a major cause of severe morbidity and, in certain conditions, high mortality, embrace a heterogeneous group of disorders characterized by an autoimmune response associated with loss of tolerance to skin adhesion molecules, leading to the formation of blisters and erosions in the skin and/or mucous membranes [1,2,3]. These skin disorders are classified into “pemphigoid diseases” and “pemphigus diseases” based on the level of blistering [2,3].

Pemphigoid diseases present with subepidermal blisters and erosions resulting from the reaction of autoantibodies directed against hemidesmosomal antigens. Among pemphigoid variants, bullous pemphigoid (BP) is the most common form and the most frequent autoimmune blistering disease [3,4,5]. BP is a highly debilitating skin disorder that typically affects the elderly and is characterized by a distinctive eosinophilic infiltration and the presence of immunoglobulin (Ig) and IgE autoantibodies to BP180 (collagen XVII) and BP230 (dystonin) [3,6,7,8,9,10]. The incidence of BP appears to be increasing over time, and estimates may greatly vary across populations, likely due to differences in underlying risk factors for BP (e.g., neurological conditions such as dementia, epilepsy, Parkinson’s disease, and stroke) and life expectancy [3,9,11]. Indeed, a meta-analysis published in 2022 reported a cumulative incidence of BP of 8.2 per million people and an incidence rate of 34.2 million person–years, with Europe as the largest contributor (10.3 per million people) [9]. BP negatively impacts the quality of life and has been associated with a three-fold increased risk of death within two years from diagnosis compared with matched disease-free controls [9,12]. Primary treatment of BP includes topical or systemic corticosteroids, depending on the extension of disease, combined with immunosuppressants or non-immunosuppressive agents, although other more specific immunomodulatory drugs (i.e., dimethyl fumarate) and new biological therapies are currently under investigation [13].

Pemphigus vulgaris (PV), pemphigus foliaceous, and paraneoplastic pemphigus are considered the main forms of pemphigus diseases. A pivotal role in their pathogenesis is represented by the development of IgG autoantibodies directed against desmosomal cadherins desmoglein (Dsg)1 and Dsg3, glycoproteins involved in the cohesion between skin keratinocytes. Autoantibodies against Dsg1 and Dsg3 induce acantholysis, i.e., loss of keratinocyte cell adhesion, which determines the formation of intraepithelial flaccid blisters [2,14,15,16,17]. In addition to being considered the most serious clinical form of pemphigus, PV is also the most widespread type, accounting for approximately 70% of cases overall [14]. Despite its development at any age, with a higher frequency recorded in children and the elderly, the onset of PV generally occurs between the ages of 40 and 60 [18]. A recent meta-analysis estimated an incidence rate of 2.83 per million person–years for PV, with no significant differences by sex, and Southern Asia showed the highest rate among the subcontinents (4.94 per million person–years) [19]. While the advent of corticosteroids in the 1950s and the subsequent increasing use of adjuvant immunosuppressants have substantially reduced mortality in PV to less than 5% [15], PV remains a life-threatening disease due to complications of therapy (severe infections, cardiovascular disease, pneumonia, septicemia, malignancies) with mortality among patients that is approximately 3 times higher than that of the general population [16,19,20]. In this context, the introduction of rituximab, the monoclonal antibody targeting the CD20 antigen of B lymphocytes, represents a promising therapeutic option for the treatment of PV and is currently recommended as the first line for new-onset moderate to severe forms [13].

Human skin represents the largest epithelial surface that can act primarily as a mechanical and microbial barrier against environmental microorganisms and harmful substances, preventing potential infections and diseases [21,22,23]. Meanwhile, with an average total mucosal surface of approximately 32 m^2^, the adult human gastrointestinal tract hosts the highest number of microorganisms, whose abundance has been estimated from 107 in the stomach up to 1014 microbial cells per gram of content in the large intestine (vs. 1012 microbial cells located in the skin) [22,24,25]. A huge number of microorganisms, including bacteria, yeasts, and viruses, collectively known as the microbiota (microbiome instead defines the total amount of microbial genomes), inhabit the human body, and the gut microbiota, composed of over 1000 species of bacteria, exerts the most relevant functions, namely nutrient extraction, metabolic regulation, immunity, and vitamin production [26,27]. The “gut–skin axis” is a new concept referring to the dual relationship between gut microbiota and skin health [28]. Therefore, the status of intestinal dysbiosis (the disruption of the balance in terms of the diversity and composition of the intestinal microbial community, which causes the “leaky gut syndrome” and the consequent deterioration of the relationship between the microbiota and the immune system) is associated with an increased risk of irritable bowel syndrome, inflammatory bowel diseases, obesity, cardiovascular disease, central nervous system disorders, cancer, and various autoimmune diseases such as rheumatoid arthritis, systemic lupus erythematosus, type 1 diabetes, celiac disease, and autoimmune thyroid disease [17,29,30,31,32]. In addition, gut dysbiosis has been shown to contribute to the pathogenesis of autoimmune dermatoses, including alopecia areata, acne vulgaris, atopic dermatitis, hidradenitis suppurativa, psoriasis, rosacea, and vitiligo [17,23,30,33]. Several immune disorders have been linked to changes in the abundance of commensal bacteria and levels of microbiota-derived metabolites such as short-chain fatty acids (SCFAs) [34]. Indeed, while under physiological conditions, SCFAs communicate with the immune system and ensure immune homeostasis, perturbations of commensal gut bacteria resulting from the disruption of the integrity of the intestinal barrier, which normally functions against pathogens and segregates microbes from host cells, increase susceptibility to infections, reduces SCFA level, lead to dysregulation of immune responses, and ultimately lead to inflammation, oxidative stress, and insulin resistance [35]. Over time, chronic intestinal dysbiosis can cause bacteria and their metabolites to cross the mucosal barrier and invade other organs, increasing the risk of various disorders [35].

On the other hand, skin dysbiosis, resulting from the invasion of bacteria and other pathogens into the deeper skin layers or even into the systemic circulation, may lead to inflammation [32]. Importantly, the microbiota is also located in three other body districts (nasal and oral cavity, vagina), of which the oral cavity represents its second largest habitat and, as such, one of the most relevant interaction windows with the external environment [36,37]. Although the study of the oral microbiota is in its infancy, increasing evidence supports the influence of oral microbes on the development of systemic diseases by promoting an inflammatory response through oral infections or the colonization of microorganisms from the oral cavity into other organs or tissues (blood, brain, gut, heart, placenta, tumors) [37].

Recently, based on the concept of the gut–skin axis, some observational studies have explored the potential association of gut, skin, and oral microbiota with BP and PV, speculating that they may act as additional risk factors and possible targets of new treatments for these autoimmune skin diseases (e.g., [17,38,39]). Therefore, in this comprehensive literature review, we summarized the up-to-date evidence on the relationship between microbiota and the onset of both BP and PV, discussing both promising and conflicting findings and plausible underlying biological mechanisms, thus paving the way for possible frontiers of future research.

## 2. The Gut Microbiota: General Features

The human gut microbiota is a complex and dynamic entity composed of approximately 100 trillion microorganisms, collectively weighing between 1 and 2 kg and encoding over 3 million genes, which are about 150 times the number of genes in the entire host genome [27,40,41]. Until a few decades ago, the properties and functions of the gut microbiota, as well as the host–microbiota interactions, were largely unknown due to limitations in examining non-cultivable microorganisms; however, recent advances in novel technologies such as 16S ribosomal RNA (rRNA) sequencing have provided an effective alternative method to microbial cultures to taxonomically characterize bacterial communities and identify the composition of the gut microbiota [42,43]. Indeed, the 16S rRNA gene encodes a highly conserved region among bacteria, which allows binding to universal primers, in addition to containing interspersed hypervariable regions that are unique to each bacterial species and thus allow their classification and characterization [40]. By targeting the 16S rRNA gene, third-generation full-length 16S rRNA amplicon sequencing technology has enabled the identification of clusters known as Operational Taxonomic Units or Amplicon Sequence Variants, whose analysis provides information on the community diversity, richness, and evenness, as well as the degree of divergence between different sample types [44,45]. In contrast to 16S rRNA sequencing, which is a cost-effective method widely employed for characterizing the profile of complex microbial communities down to the genus level, metagenomic sequencing collects information on both the taxonomic composition and functional genes of ecosystems by sequencing the genomes of all species isolated from the entire microbial community [46,47].

It has been estimated that up to 90% of the gut microbiota is represented by Firmicutes and Bacteroidetes, with the Firmicutes phylum being the most abundant, comprising more than 200 different genera and composed mainly of gram-positive bacteria (*Clostridium*, *Lactobacillus*, *Bacillus*, *Enterococcus*, and *Ruminococcus*), while Bacteroidetes are gram-negative bacteria primarily consisting of *Bacteroides* and *Prevotella* [48,49]. On the other hand, Actinobacteria, Proteobacteria, Fusobacteria, and Verrucomicrobia represent most of the residual portion of the intestinal microbial composition [49]. It is worth noting that the composition of the intestinal microbiota changes taxonomically not only throughout the gastrointestinal tract but also with gestational age, mode of delivery, feeding methods, and antibiotic treatment [48]. Additionally, the gut microbiota profile remains relatively stable throughout adulthood, although it may be subject to variations depending on age (people over 70 years of age can be affected by problems with digestion and nutrient absorption), lifestyle (medications, smoking, physical exercise, mental health), diseases (intestinal and extra-intestinal disorders), and, mainly, diet (both habitual diet and short-term dietary changes determine variations in the composition of the gut microbiota) [41,49,50]. Conversely, variations in the gut microbiota between individuals can be attributed to differences in enterotypes (individual-specific clusters of bacteria), ethnicity, body mass index, frequency of exercise, and dietary and cultural habits [48].

**Figure 1:**
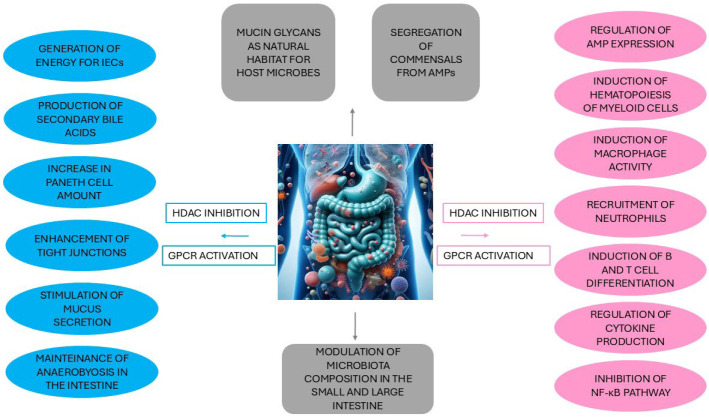
Schematic representation of the complex relationship between intestinal epithelium cells, the immune system, and the gut microbiota. The effects of gut microorganisms on the intestinal epithelium (light blue) and on the innate and adaptive immune systems (pink) are expressed through the inhibition of histone deacetylase activity and the activation of G protein-coupled receptor signaling. The actions of intestinal epithelial cells and immune cells on the microbiota are shown in grey. Image partially generated using the Microsoft Bing Artificial Intelligence tool (see text for more details). Abbreviations: AMP: antimicrobial peptides; GPRC: G protein-coupled receptor; HDAC: histone deacetylase; IECs: intestinal epithelium cells; NF-κB: nuclear factor kappa-light-chain-enhancer of activated B cells.

The intestinal microbiota has multiple properties, e.g., protection, metabolic activities, and regulation and development of the immune system, as described below (see also Figure 1), through which it establishes close crosstalk with the intestine, influencing the health–disease status and the subsequent functioning of all organ systems (reviewed in [27,30,51,52]).

### 2.1. The Crosstalk Between the Gut Microbiota and Intestinal Epithelial Cells

The gut microbiota interacts with the intestinal epithelium, which consists of different populations with specific functions: columnar cells, which participate in the digestion process; Goblet cells, producing mucus and, by presenting antigens to dendritic cells located in the lamina propria, contribute to the formation of immune tolerance; Tuft cells, which participate in the removal of parasites from the intestinal lumen by producing interleukin (IL)-25; Paneth cells that regulate microbial diversity in the intestine through the secretion of antimicrobial peptides (AMPs) and growth factors; enteroendocrine cells producing peptides and hormones that stimulate peristaltic movements; microfold cells, which stimulate the immune system by binding antigens that are subsequently transported to dendritic cells; dendritic cells, which can promote an antigen-specific immune response by interacting with T cells; and B cells, which regulate the gut microbiota through IgA secretion [27,53].

Overall, intestinal epithelium cells (IECs) form a cytoskeleton structure composed of tight and adherence junctions and desmosomes located along the lateral membrane, which confers mechanical strength between cells and ensures impermeability to the gut barrier, preventing the translocation of microorganisms from the intestinal lumen to the deep tissues [54]. Indeed, the intestinal mucus layer, which has the primary role of preserving intestinal homeostasis and whose thickness varies in the intestine (much higher in the large intestine than in the small intestine due to the density of microorganisms), is implicated in regulating the delivery of nutrients and drugs and protecting the host from mechanical attacks and food toxins [27,55]. The relationship between IECs and microbiota is bidirectional. In fact, while the microbiota contributes to the integrity of the intestinal barrier by participating in the stimulation of mucus secretion, the mucus layer, which contains mucin glycans that act as attachment sites and nutrients, represents a natural habitat for microorganisms and can also influence the composition of mucus-associated bacteria thanks to the glycosylation profile of mucin [54,55,56].

The gut microbiota produces a series of metabolites having various functions in humans, some of which are identified as playing key roles in modulating IEC activity and preserving the gut epithelium barrier [57]. The bacterial fermentation of dietary fibers in the large intestine produces SCFAs, a group of organic compounds with less than six atoms of carbon mainly produced by Firmicutes and Actinobacteria and including acetate, propionate, and butyrate with a molar ratio of around 65, 20, and 15%, respectively [27,48,53,58]. Despite its lower abundance among SCFAs produced, butyrate is the preferred fuel source for IECs, contributing to 60–70% of their energy requirements, thereby promoting tight junction integrity, cell proliferation, and mucus secretion [59]. SCFAs amount reflects a healthy gut microbiota. Indeed, butyrate, used by colonocytes to generate energy, causes oxygen consumption that, by creating an anaerobic intestinal environment, prevents the invasion of pathogenic bacteria such as *Escherichia coli* and *Salmonella* [52]. The amount of SCFAs can also control central appetite (primarily acetate), reduce weight gain (acetate and propionate), and regulate glucose and lipid metabolism, inhibiting hepatic gluconeogenesis (propionate), reducing lipogenesis (acetate), and increasing the secretion of leptin (butyrate), a hormone that plays a fundamental role in energy balance and body adiposity [50,58,60].

Together with SCFAs, gram-positive bacteria such as *Bifidobacterium*, *Clostridium*, *Enterococcus*, and *Lactobacillus*, and gram-negative bacteria belonging to *Bacteroides* can synthesize secondary bile acids (deoxycholic acid and lithocholic acid—LCA) from primary bile acids (BAs) [27,61]. BAs such as chenodeoxycholic acid and cholic acid, metabolites secreted by the liver, play a primary role in the digestion and absorption of lipids and the uptake of cholesterol and fat-soluble vitamins [62,63]. Approximately 95% of BAs are reabsorbed by IECs and then excreted in the feces through deconjugation, dihydroxylation, and oxidation, while unabsorbed bile acids act as substrates for gut microbial metabolism [27,57,62,63].

Recently, the gut microbiota has also emerged as a master regulator of tryptophan metabolism, an essential amino acid that is metabolized through three major pathways: (i) primarily through the kynurenine pathway, leading to the generation of kynurenine and other related compounds; (ii) conversion, to a lesser extent, into various indole derivatives; and (iii) the serotonin pathway for the synthesis of serotonin and melatonin [26]. Tryptophan-derived gut metabolites contribute to the maintenance of the gut barrier, while recent evidence indicates that, like butyrate, indole activates the aryl hydrocarbon receptor in IECs, leading to the enhancement of tight junctions [64,65]. Serotonin, a neurotransmitter primarily synthesized in the enterochromaffin cells (a subtype of enteroendocrine cells) of the gut, has also been reported to modulate gut microbiota composition [66]. At the same time, serotonin synthesis is, in turn, regulated by the relative levels of SCFAs, particularly butyrate [67].

### 2.2. Interaction Between the Gut Microbiota and Immunity

The gut microbiota establishes a close dual interplay with the innate immune system, and since this interaction is highly complex and dynamic, any perturbation in the composition of the gut microbiota or host–microbiota interfaces or dyshomeostasis in the immune system can potentially cause systemic dissemination of commensal microorganisms, increased susceptibility to infections, and an exaggerated immune response [68]. AMPs, a class of diverse compounds produced by Paneth cells and immune cells in the gastrointestinal tract, are key effectors of innate immunity, playing crucial roles both in the host defense against enteric infections and in the maintenance of immune tolerance to the gut microbiota [69]. While SCFAs regulate AMP expression, AMPs can modulate the composition of the gut microbiota, preserving species–specific bacterial communities [69,70]. Furthermore, Paneth cells, through the production of AMPs, promote the regeneration of RegIIIγ, a secreted antibacterial lectin, and the consequent segregation of the microbiota and IECs, avoiding potentially harmful and unnecessary immune responses [57,71]. SCFAs exert their effects on both the epithelial barrier function as well as on mucosal and systemic immunity through the direct inhibition of histone deacetylases, a class of enzymes capable of removing acetyl groups from histones and other protein regulatory factors [34,72]. SCFAs may also activate G protein-coupled receptors (GPCRs) GPR41, GPR43, and GPR109A, which belong to one of the largest families of proteins that feature a conserved structure composed of seven transmembrane helices [34,52]. In particular, GPCR43 is a major target of SCFAs for the regulation of the immune system and control of inflammation, helping to prevent the development of various chronic inflammatory diseases [73].

Gut-associated lymphoid tissues (GALT), which histologically embrace the appendix, crypt patches, isolated lymphoid follicles, mesenteric lymph nodes, and Peyer’s patches, represent the critical link between the non-specific immune response to the gut microbiota and the subsequent adaptive immune response, thus playing a pivotal role in maintaining homeostasis between gut microorganisms and the immune system [51]. GALT is based on the recognition of pathogen-associated molecular patterns (PAMPs) on enteric bacteria via various pattern recognition receptors (PRRs), such as Toll-like receptors (TLRs) (located on the cell membrane and able to recognize exogenous PAMPs) and nucleotide-binding oligomerization domain (NOD)-like receptors (NLRs) (cytosolic receptors specialized in defense against intracellular pathogens), and the activation of a downstream signaling cascade, which in turn leads to the secretion of cytokines (IL-1β, IL-6, IL-12, IL-18) and chemokines (tumor necrosis factor-alpha—TNF-α, CXCL8, and CCL10) [27,51,68,74]. Like AMPs in the small intestine, this mechanism serves as a major regulator of the microbiota in the large intestine [27]. TLRs, and in particular TLR5, in addition to being involved in host defense against pathogens, regulate the abundance of commensal microbes, while, conversely, TLR5 deletion causes microbial dysbiosis [75]. Similarly, myeloid differentiation primary response gene-88 (MyD88), an adapter protein localized on TLRs, regulates T cell differentiation and also promotes microbial homeostasis by stimulating IgA secretion, while its suppression is associated with an altered microbial composition [74,76].

Notably, some NLRs give rise to multiprotein complexes known as inflammasomes, which, through cleavage and activation of caspase-1, lead to the activation of pro-IL-1β and pro-IL-18 in mature forms that, in turn, preserve the homeostasis of the gut microbiota [77]. NOD-pyrin domain-containing 6 (NLRP6), in particular, has been associated with the modulation of colonic microbial ecology and intestinal balance [68,78].

Dendritic cells, monocytes, and macrophages, other key effectors in the innate immune response and distributed within the epithelium and intraepithelial layers, lamina propria, and structures of GALT, exert crucial functions of phagocytosis and antigen presentation to the adaptive immune system and are also involved in maintaining immune tolerance to commensal bacteria [51]. On the other hand, SCFAs can promote hematopoiesis of myeloid cells in the bone marrow and polarize macrophages through epigenetic modulation, as well as trigger different local immune responses of dendritic cells [51]. In the context of anti-inflammatory properties, SCFAs can also increase the production of antimicrobial peptides (e.g., calprotectin) by macrophages and regulate the chemotactic recruitment of neutrophils through both inhibition of HDAC activity and GPR43 signaling [34].

Although less explored until recent years, accumulating evidence suggests the existence of a mutualistic relationship also between the intestinal microbiota and the adaptive immune system. Indeed, B cells produce a large array of IgA antibodies responsive to commensals [68], while the gut microbiota and SCFAs induce B cell differentiation and intestinal IgA secretion [34]. SCFAs also induce differentiation of T regulatory cells via both HDAC inhibition and the GPR43 pathway [34]. In particular, butyrate favors the differentiation of naïve T cells to effectors such as T helper (Th) 1 and Th17 cells, subsets of CD4^+^ regulatory T cells that, by releasing cytokines like IL-2, IL-17A, IL-17F, IL-21, IL-22, TNF-α, and interferon-gamma (IFN-γ), promote the survival, differentiation, and antitumor activities of CD8^+^ cytotoxic cells, the most powerful effectors against intracellular pathogens and cancerous cells [68,79,80]. Additionally, SCFAs have anti-inflammatory effects by influencing cytokine expression in T cells [34]. Butyrate induces the upregulation of IL-10 and transforming growth factor-beta and suppresses the production of proinflammatory cytokines, i.e., IL-1β, IL-6, IL-8, TNF-α, and IFN-γ, through the inhibition of the nuclear factor kappa-light-chain-enhancer of activated B cells (NF-κB), a transcription factor involved in the regulation of immune responses, inflammation, cell proliferation, survival, and apoptosis [81,82].

## 3. The Gut–Skin Axis

**Figure 2:**
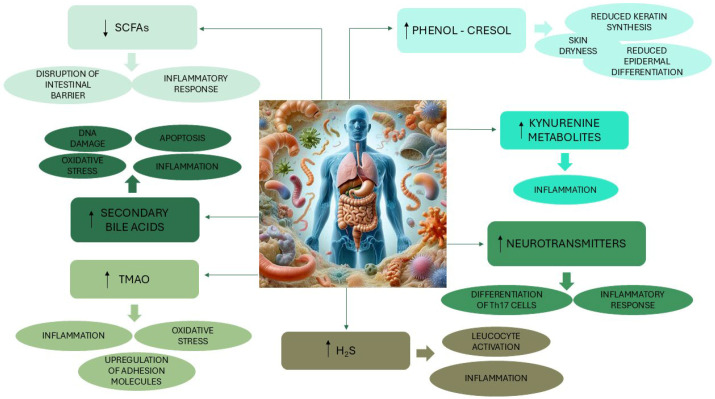
Summary of the biological effects produced by intestinal microbiota metabolites in a state of dysbiosis and potentially associated with the onset of inflammatory skin diseases (see text for more details). Vertical arrows indicate increased or decreased effector levels. Image partially generated using the Microsoft Bing Artificial Intelligence tool. Abbreviations: H_2_S: hydrogen sulfide; SCFAs: short fatty acids; Th: T helper cell; TMAO: trimethylamine-N-oxide.

Based on growing evidence suggesting that alterations in the gut microbiota may promote the development of diseases outside the gastrointestinal tract, including atherosclerosis, cardiometabolic diseases, hypertension, chronic kidney diseases, obesity, type 2 diabetes, and even inflammatory skin disorders, the concept of a gut–skin axis has also been postulated [25,83,84]. The gut microbiota composition appears to be associated with the development of psoriatic disease, with an increased F/B ratio, which could reflect the association of psoriasis with metabolic and cardiovascular comorbidities [85]. A non-negligible number of immune-mediated diseases, including psoriasis and pyoderma gangrenosum, are more frequently diagnosed among patients with inflammatory bowel disease, a group of disorders represented mainly by ulcerative colitis and Crohn’s disease, which result from interactions between genetic susceptibility, environmental triggers, and lifestyle/dietary factors [86,87]. Furthermore, atopic dermatitis has been associated with intestinal dysbiosis characterized by reduced microbial diversity and a decrease in commensal microbes like *Lactobacillus* and *Bifidobacterium*, which leads to an enhanced inflammatory response in the skin [88]. Likewise, in acne, a decrease in *Cutibacterium* in the skin is concurrent with reduced microbial diversity in the gut, which in turn may worsen the clinical severity of the cutaneous disease [88]. The connection between the skin and the gut appears to be mediated by the activities of immunological components and inflammatory mediators located between the two districts, as well as by neuroendocrine signaling [30,88]. While the microbiota is a key modulator of the immune system, the presence of pathogens or other host-specific factors (lifestyle, diet, antibiotics) can influence the composition and function of the gut microbiota by activating the immune system, thus leading to a decrease in the diversity of the microbial community, a loss of commensals, or an overgrowth of harmful microorganisms [22,30,89]. A decrease in SFCA producers caused by dysbiosis results in a proinflammatory state of the gut that affects the production of the mucus layer [30,84]. The increased permeability of the gut barrier facilitates the passage of microorganisms, together with their toxic metabolites and neurotransmitters, into the circulatory system and then into a variety of organs, including the skin [30,90] (Figure 2). In fact, SCFAs are believed to influence the predominance of certain skin microbiotic profiles, which subsequently induce cutaneous immune responses [90]. Furthermore, reduced diversity and richness of the gut microbiota, such as those that occur following a high-fat, low-fiber “Western diet”, are often related to alterations in the composition of the secondary BA pool that participates in the enterohepatic cycle and is involved in the immune–inflammatory response [91,92,93]. Thus, while at relatively low concentrations (e.g., <50 μM) secondary, BAs exert anti-inflammatory actions by primarily binding to the G protein-coupled bile acid receptor 1 (TGR5) in various organs, higher concentrations of these molecules (up to 1 mM) lead to detrimental effects on the intestinal epithelium, activating the NF-κB and the extracellular signal-regulated kinase 1 and 2 signaling pathways (cascades regulating cell proliferation, differentiation, migration, metabolism, and survival) and inducing the generation of reactive oxygen species, mutations, DNA damage, and apoptosis [61,94,95]. Patients with rosacea present an increase in circulating secondary BAs, particularly LCA, which, by activating the expression of TGR5 in keratinocytes, promotes the production of cytokines and chemokines [92].

Similarly, diet, gut dysbiosis, or changes in the composition of the intestinal microbiota (i.e., a higher Firmicutes/Bacteroidetes—F/B ratio, recognized as a marker of normal/compromised intestinal homeostasis [96]) or damage to the intestinal barrier may lead to increased levels of trimethylamine-N-oxide (TMAO), synthesized in the liver from trimethylamine, a molecule resulting from the metabolism of phosphatidylcholine/choline, carnitine, betaine, dimethylglycine, and ergothioneine contained in dietary compounds, and mainly produced in the colon by *Clostridia*, *Shigella*, *Proteus*, and *Aerobacter* [97,98]. An increase in circulating TMAO triggers the activation of the NF-κB pathway and NLRP3 inflammasome, which plays a complex role in the innate immune response and inflammatory signaling [99]), upregulates the expression of inflammatory cytokines, adhesion molecules, and chemokines, and induces oxidative stress [100]. TMAO is indeed considered a marker of bacterial translocation and systemic inflammation and a prognostic factor for atherosclerosis, cardiovascular disease (e.g., heart failure, recurrent ischemic events), metabolic syndrome, renal failure, and inflammatory skin diseases such as hidradenitis suppurativa, an auto-inflammatory skin disease generally associated with cardiovascular and metabolic comorbidities [83,101,102,103].

Dysbiosis of the gut microbiota causes alterations in the relationship between microbial tryptophan metabolism and the host intestinal immune system, resulting in dysregulation of kynurenine and serotonin pathways [26]. Disruption of tryptophan catabolism pathways may ultimately contribute to the development of inflammatory conditions, autoimmune diseases, metabolic syndrome and other cardiovascular comorbidities, kidney disease, neuropsychiatric disorders, and cancer [26,104]. Although with conflicting results, levels of metabolites and enzymes of the kynurenine pathway are positively associated with psoriasis, probably due to an increased production of cytokines IL-17 and IL-22, leading to skin proliferation and development of psoriatic lesions [104].

Disturbances in gut microbiota diversity also affect the production of neurotransmitters (e.g., γ-aminobutyric acid (GABA), serotonin, dopamine, and noradrenaline) [30]. Altered levels of neurotransmitters, in addition to being linked to a broad spectrum of immune-related neurological disorders, including developmental and psychiatric diseases and neurodegeneration, may also adversely influence the physiological functioning of the skin via the nervous system [30,105,106]. Indeed, binding of neurotransmitters to receptors expressed on immune cells located in the dermis and epidermis modulates the immune system response, promoting an inflammatory process characterized by differentiation of Th17 cells and secretion of proinflammatory molecules (e.g., IL-1, IL-6, IL-23, IFN-γ, TNF-α), cell migration, vascular permeability, and mast cell degranulation, which ultimately may result in the onset of skin diseases, such as psoriasis [84,107,108].

Free phenol and p-cresol metabolites of aromatic amino acids produced by certain pathogenic bacteria like *Clostridium difficile* in conditions of disturbed gut environment may enter the circulation and accumulate in the skin, where in vitro, they reduce the production of keratin, thereby affecting epidermal differentiation and skin barrier integrity [90,109]. A positive association of cresol levels with skin dryness and disruption of keratinization was also reported in adult women, where the daily use of prebiotics and probiotics reduced serum total phenol levels and restored healthy skin [110].

Finally, excessive production of hydrogen sulfide (H_2_S), recognized as a gaseous signaling molecule having anti-inflammatory functions at physiological levels, can negatively affect the intestinal mucosa, leading to alterations of the relative abundance, functions, and spatial organization of intestinal bacteria and disruption of mucus integrity through the breaking of mucus disulfide bonds [111,112]. H_2_S is produced from sulfur-containing dietary amino acids (e.g., cysteine, methionine, taurine) by sulfate-reducing bacteria in the gut and achieves its highest concentration in the colon (250 mM) [113,114]. Most of the effects of H_2_S are highly concentration-dependent, and while in normal skin conditions, it regulates vasodilatation and apoptosis, promotes keratinocyte differentiation, modulates pruritus, and suppresses inflammation, alterations in H_2_S production, induces the production of proinflammatory mediators and leukocyte activation, contributing to the pathogenesis of some skin disorders (psoriasis, melanoma, and other dermatoses) [115].

Although the association between gut and skin microbial communities is an emergent area of research and the mechanisms by which the gut microbiota interfere with skin balance remain to be fully elucidated, current data on potential biological drivers involved in the gut–skin axis as well as skin disease risk (represented in Figure 2) indicate the direction for future studies to further investigate this relationship in order to promote both gut and skin health.

## 4. The Gut Microbiota and the Relationship with Autoimmune Bullous Diseases

As described in the previous section, gut microbiota dysbiosis can induce both local and systemic immune responses, thus potentially contributing to the development of autoimmune diseases, which, in addition to being considered among the major causes of morbidity and mortality, are collectively characterized by long-lasting debilitating symptoms, organ impairment, and high treatment costs [86,116,117]. The burden of BP and PV is substantial due to a variety of comorbidities due to viral and bacterial infections or long-term corticosteroid use, which result in high rates of hospitalization and increased risk of mortality [118,119]. Therefore, a deeper understanding of modifiable risk factors, including the composition and diversity of the gut microbiota, can help in discovering more effective and targeted treatments to improve the quality of life of patients with debilitating diseases.

### 4.1. The Association of the Gut Microbiota with Bullous Pemphigoid

The pathophysiological hallmark of BP is the production of autoantibodies directed against two structural components of hemidesmosomes (a highly specialized multiprotein complex that ensures firm adhesion between basal epithelial cells and the dermal extracellular matrix), BP230 and BP180 [120,121]. While genetic variations in the human leukocyte antigen system (encoding proteins involved in immune regulation) have been established as the most significant predisposing factors in BP, contributing to the loss of immune tolerance towards the two hemidesmosomal anchoring proteins, the imbalance between Th and Treg cells, the activation of toll-like receptors and of Th2- and Th17-related cytokines cascades, the latter maintaining the inflammatory response by inducing cytokine production, are responsible for antibody secretion in BP [4,121,122,123].

To date, four studies have examined the gut microbiota’s impact on the development of BP (Table 1). The pilot study by Scaglione et al. [38] reported an underrepresentation of Proteobacteria in the gut microbiota of BP patients, with Firmicutes and Bacteroidetes as the most abundant phyla, reflecting a similar composition to that of healthy subjects and with no significant differences from PV patients. In the study by Liu et al. [10], who recruited 66 pairs of BP patients and their controls matched for age, sex, and study center, participants were further sub-grouped by patients’ disease status (first diagnoses and relapsed cases) to assess the relationship between gut dysbiosis and BP disease activity. The authors observed both decreased richness and evenness as well as alterations in the composition of the gut microbiota in BP patients [10]. An enrichment of *Flavonifractor* and *Flavonifractor plautii* was detected in BP patients and relapsed cases, respectively, supporting the hypothesis that these microbial species can be associated with oxidative stress and inflammation, probably due to their ability to degrade beneficial anticarcinogenic flavonoids [10,124]. However, the enterobacterium *Flavonifractor plautii* can also abolish antigen-induced Th2 immune responses and attenuate inflammation by suppressing TNF-α expression and IL-17 signaling in the adipose tissues and intestine, respectively, leaving doubts about its real function [125,126,127]. In contrast, BP patients exhibited a reduced abundance of *Faecalibacterium prausnitzii*, a well-known butyrate-producing bacterium belonging to Firmicutes, which, thanks to the properties of its metabolites butyrate and salicylic acid, can inhibit IL-8 production by suppressing the NF-kB pathway, upregulate Treg cell production, and induce IL-10 secretion, thus blocking the synthesis of pro-inflammatory cytokines IL-6 and IL-12 and, in general, inflammatory processes [128,129]. Lower amounts of *Faecalibacterium prausnitzii* were observed in patients with ulcerative colitis and Crohn’s disease [130,131] and psoriasis [84], while *Faecalibacterium prausnitzii* as a probiotic supplement can be used in the prevention and management of colorectal cancer [132] and type 2 diabetes [133]. Furthermore, shotgun metagenomic sequencing of the gut microbiome revealed significant alterations in twelve gut microbiota pathways in BP patients, nine of which were significantly increased, i.e., those related to pyridoxal 5′-phosphate biosynthesis, fatty acid biosynthesis, and biotin biosynthesis [10]. Pyridoxal 5′-phosphate acts as a cofactor of glutamate decarboxylase (GAD) in the conversion of L-glutamate into GABA, a primary inhibitory neurotransmitter that is also a signaling molecule produced by bacteria, fungi, plants, and invertebrates [134]. The authors identified in BP patients both the GABA shotgun, a three-step pathway that provides an alternative route for the synthesis of succinate from α-ketoglutarate in the mitochondrial-based tricarboxylic acid cycle for subsequent bacterial energy production, and the biosynthesis of putrescin, which, like ornithine and arginine, is an alternative precursor of GABA [134,135]. Putrescine, a major metabolite of gut bacteria that can promote a “leaky gut” condition during both intestinal autoinflammation and infection [136], is positively correlated with the expression of inflammatory chemokines in patients with psoriasis [137]. Additionally, putrescine, being involved in several cellular processes (proliferation, stress protection, metabolism, and regulation of the immune response), like other polyamines, if dysregulated, can impact growth, aging, and diseases such as cancer, metabolic disorders, and neurodegeneration [138,139]. Although the reported biological activities of GABA include alleviation of pruritus, attenuation of atopic dermatitis lesions by promoting Th1 response, increased expression of type 1 collagen, and maintenance of skin elasticity, the GABA shunt appears to play a key role in BP, consistent with the significantly positive association of GABA biosynthesis from putrescine with atopic dermatitis severity [25,30,134,140]. These inconsistent results could be attributed to differences in the components and activities of GABA shunt bacterial species even within the same genus, with some bacteria containing multiple GAD enzymes and others containing no GAD homologs at all [10,140]. Another Chinese study [39] recruiting consecutive patients diagnosed with BP, further divided into those in the active stage and in the remission stage, documented an increased trend of alpha diversity (a score referring to the species richness or evenness within a functional community [141]) in patients with BP onset compared with the control group, unlike the study of Liu et al. [10]. This finding could be explained by the enrichment of pathogenic bacteria in patients with active BP, namely Bacteroidaceae and Ruminococcaceae, and the reduced relative abundance of Lachnospiraceae compared with healthy controls [39]. Ruminococcaceae, among the most widespread families of Firmicutes, is one of the few taxa that include secondary BA producers, known to cause pro-inflammatory effects [142], although its relationship with immune-mediated diseases is somewhat controversial, with a low relative abundance associated with inflammatory bowel disease [143] and immunoglobulin E-associated eczema [144] and higher relative abundance correlated with asthma [145] and reactive arthritis [146]. Conversely, members of Lachnospiraceae, including *Blautia*, *Coprococcus*, *Lachnospira*, *Roseburia*, *Ruminococcus*, are massive producers of SCFAs and, as such, may alleviate inflammatory and allergic diseases by exerting anti-inflammatory and immunomodulatory effects [147,148]. Therefore, reduced levels of Lachnospiraceae can lead to an imbalance in the functionality of the gut microbiota, resulting in an increased risk of inflammatory skin diseases such as alopecia areata [149], hidradenitis suppurativa [150], and PV [151]. Patients with active BP also showed a reduced proportion of Prevotellaceae but a higher proportion of *Prevoltella copri* [39]. Relative abundance of Prevotellaceae is linked to the dietary habits of rural and preagricultural or isolated populations, and if it may improve cardiovascular risk and glucose metabolism [151], it also includes strains such as *Prevotella copri*, a frequent inhabitant of the gut microbiota, which also shows pathobiontic properties such as releasing inflammatory mediators [152,153]. *Prevotella copri* is, in fact, associated with both inflammatory conditions, including rheumatoid arthritis [154], human immunodeficiency virus-1 infection [155], insulin resistance [156], ankylosing spondylitis [157], and metabolic benefits by improving glycemic control [158], depending on dietary habits [159,160,161]. Active disease subjects were also characterized by a depletion of *Vellonella dispar*, a Gram-negative commensal bacterium that produces SCFAs by lactate fermentation [162], and *Bacteroides ovatus*, a commensal microbe that, due to its ability to consume a variety of food sources, colonizes the intestine, helping to prevent pathogen invasion and related inflammation [163]. Furthermore, some species shared significant correlations with clinical parameters [39]. Notably, the composition of the gut microbiota in BP patients in remission resembled that of the control group, indicating that the treatment can reduce harmful bacteria that cause inflammation, further emphasizing the involvement of intestinal microbial communities in disease development [39]. In assessing the gut microbiota profile in BP patients, Han and coauthors [164] found a higher relative abundance of pathogens and a reduction in probiotics in affected subjects, who exhibited a significant difference in beta diversity (an index describing the amount of overlap or differentiation between species communities [141]) compared with controls and an overall worse dysbiosis than that observed in PV patients. More specifically, subjects with BP presented an enrichment of Proteobacteria, although the predominance of this phylum was less evident than in PV patients, as previously observed by Scaglione et al. [38]. Furthermore, in line with the above-mentioned findings [10,39], the authors observed an increased abundance of Bacteroides and *Prevotella* and a reduction in *Faecalibacterium*, the latter being strictly and negatively correlated with autoantibody titer [164]. Functional analysis revealed significantly increased alterations in BP patients compared with PV patients in certain microbial pathways, i.e., *Escherichia coli* infection, shigellosis, bacterial invasion of epithelial cells, and biosynthesis of lipopolysaccharide (LPS, a vital cell wall constituent of Gram-negative bacteria that can directly modulate the immune system by inducing cytokine production [165]), suggesting a different role of gut microbiota in bullous diseases [164].

In summary, despite the limited number of investigations, the small sample size, and the lack of longitudinal data that generally characterize the studies analyzed, as well as the presence of only one study using shotgun metagenomic sequencing to explore gut microbiota function in BP, there are signals of association between gut microbiota dysbiosis and BP pathogenesis, with alterations in relative abundance in certain identified taxa (i.e., drop in *Faecalibacterium prausnitzii*, increase in *Prevotella copri*) and metabolic routes (i.e., GABA shunt and related pathways and functional and inflammatory pathways) potentially involved in the inflammation process within the gut–skin axis. Furthermore, the different profiles of the intestinal microbiota at different stages of the disease suggest the possibility of supplementation with prebiotics, probiotics, and symbiotics to improve intestinal microbiota homeostasis, preserve the integrity of the intestinal barrier, regulate the immune response, and facilitate disease remission.

### 4.2. The Association of the Gut Microbiota with Pemphigus Vulgaris

PV is an autoimmune disease characterized primarily by the presence of IgG antibodies against Dsg1 and Dsg3 due to the involvement of immune responses mediated by B cells (crucial to producing specific autoantibodies) and T cells (participating in the onset and persistence of PV) [153,166]. In particular, Th1 and Th17 appear to play a predominant role in PV pathogenesis, with PV patients exhibiting higher levels of inflammatory Th1 (IL-1RA, IL-1β, IL-2, IL-12p70, GM-CSF, TNF-α, IL-18) and Th17 cytokines (IL-17, IL-22, IL-23) than healthy controls [166,167].

In recent years, a few studies have explored the association between gut microbiota status and PV (Table 2). In an Italian pilot study aimed at evaluating potential microbial alterations in the gut, skin, and oral cavity of PV subjects, gut microbiota analysis revealed a similar composition at the phylum level to that of healthy subjects, with Firmicutes showing the highest relative abundance, followed by Bacteroidetes and Proteobacteria [38]. Huang et al. [153] first compared gut microbiota in PV patients and healthy controls also with regard to circulating cytokines of Th1/Th2/Th17 cells. Although the two groups showed no significant differences in alpha diversity, analysis of beta diversity revealed that, at the genus level, PV patients had reduced levels of *Lachnospiracea_incertae_sedis* and *Coprococcus*, whose decline in the gut microbiota leads to reduced production of SCFAs and has been related to the development of allergies, asthma, inflammatory bowel disease, metabolic disorders, and mental and neuropsychological disorders [137,153,168,169,170]. The case group was characterized by increased levels of *Granulicatella* and *Flavonifractor*, the latter not only linked to different conditions such as affective disorders and colorectal cancer but also associated with oxidative stress and increased inflammation, and whose higher relative abundance was also observed in BP patients [10,153,171,172]. Consistently, Huang and coworkers [153] found that *Flavonifractor* was positively correlated with circulating levels of C5a, IL-1β, IL-6, IL-7, IL-8, and IL-21, while *Lachnospiracea_incertae_sedis* and *Coprococcus* were both negatively associated with IL-17A, indicating that changes in the gut microbiota may induce an unbalanced Th1/Th2 or Th17/Treg differentiation and related abnormal cytokine production, typical of PV. Guo et al. [173] reported that the gut microbiota of patients with PV had lower diversity and richness than those of healthy controls, and the two microbial communities showed a significant degree of dissimilarity. Indeed, although the F/B ratio was not significantly different between the two groups, the gut microbiota of PV patients presented an increased abundance of Proteobacteria and Verrucomicrobia and a depletion of Firmicutes, which include producers of SCFAs such as Lactobacillaceae, Ruminococcaceae, and Lachnospiraceae [52,173]. Consequently, the abundance of bacteria like *Butyricicoccus*, *Clostridium*, *Megamonas*, *Roseburia*, *Faecalibacterium prausnitzii*, and *Lactobacillus murinus* was significantly reduced in these subjects [173]. In contrast, pathogenic bacteria such as *Escherichia coli*, *Klebsiella*, *Bacteroides fragilis*, *Enterobacter hormaechei*, and *Shigella pneumoniae* were considerably enriched in patients with PV and also showed internal positive correlations, thus resulting in increased biosynthesis of LPS [173]. These opportunistic bacteria were also positively associated with anti-Dsg1 and anti-Dsg3 antibody levels and with the Pemphigus Disease Area Index, a validated score used to assess the severity of PV [173,174]. Additionally, metabolomic analysis on stool samples revealed abnormal accumulation of lipids and lipid compounds, especially phosphatidylethanolamine, the second most abundant class of glycerophospholipids whose metabolism dysregulation can promote inflammasome activation and contribute to the development of chronic inflammatory diseases [173,175,176]. Of note, phosphatidylethanolamine content was positively correlated with *Streptococcus parasanguinis* and *Klebsiella pneumoniae* and negatively correlated with *Roseburia* spp., suggesting the potential involvement of these microbial species and their metabolites in the pathogenesis and progression of PV [173]. Another case-control study documented significant differences in the composition, but not in the richness, of the gut microbiota species in PV patients compared with healthy family members [117]. In particular, the cases exhibited an enrichment of *Escherichia coli*, which, being a Gram-negative bacterium, is coated with LPS and therefore able to induce an inflammatory response and, by creating a “leaky gut”, to trigger the onset of autoimmune diseases [117,177]. These subjects also had a concomitant decrease in the relative abundance of probiotics and certain SCFA-producing species, which is likely attributable to the inhibitory action of *Escherichia coli* via the production of toxins and enterobactin (the latter a conserved siderophore that chelates iron from iron-binding host proteins, suppressing the growth of other bacteria [178]). *Escherichia coli* may also exacerbate inflammation and disease progression due to the lower levels of SCFAs found in PV patients, although they were partially restored after one month of glucocorticoid therapy [117]. Although glucocorticoid treatment did not significantly change alpha and beta diversity in PV patients, it caused a decrease in the relative abundance of *Escherichia coli* in responders, indicating that this therapy may help restore intestinal homeostasis [117]. In PV patients, *Escherichia coli* displayed a strong correlation with the phosphotransferase system pathway, a conserved cascade involved in the transport and phosphorylation of selected carbohydrates as well as in the virulence of several pathogenic bacterial species, suggesting that this pathway, enriched in these patients, may contribute to the toxic effects of *Escherichia coli* [117,179]. Confirming previous results, Li et al. [17] observed no significant differences in alpha diversity of the gut microbiota when comparing patients with pemphigus in an active stage (75% of whom had PV), patients with pemphigus in remission stage characterized by the absence of new lesions (63.7% of them had PV), and healthy controls. The authors also found no significant alterations in the composition of the microbial communities between the three groups, in line with the study by Wang et al. [117]. Conversely, the relative abundance of gut microbiota taxa in pemphigus patients was altered compared with controls [17]. Indeed, the active pemphigus group presented an enrichment of Bacteroidetes and Proteobacteria and a depletion of Firmicutes, with a slight decrease in the F/B ratio and a significantly higher proportion of pathogenic bacteria than the pemphigus remission group, as previously reported by Guo et al. [173], supporting the involvement of gut microbiota dysbiosis in pemphigus development [17]. Furthermore, specific taxa were correlated with autoantibody titers. In particular, *Veillonella*, which has been reported to reduce the risk of asthma and bronchiolitis by inducing a mixed Th1/Th2/Th17 lung inflammatory response [180,181], was depleted among patients with active pemphigus, thus explaining the observed inverse correlation with anti-Dsg3 antibody titers [17]. In contrast, the abundance of *Prevotella*, which was lower in these patients than in the other groups, as observed in subjects affected by BP [39], was positively associated with anti-Dsg1 antibody titers [17]. The effects of the *Prevotella* genus (members of which are anaerobic Gram-negative bacteria of the Bacteroidetes phylum) on the host are still debated [166]. As discussed in Section 4.1, *Prevotella* strains are generally considered commensal bacteria, with increased abundance (linked to a diet rich in non-starch polysaccharides, resistant starch, and fiber content) associated with improved glucose metabolism [159,182] but in a state of dysbiosis they may cause localized infections and inflammatory diseases such as periodontitis due to the production of virulence factors (e.g., LPS, hemolysins adhesins, proteases), which enhance the proliferation and survival of microorganisms [183]. Coriobacteriaceae that, as SCFA producers, contribute to the homeostasis of intestinal microbiota, preserving the integrity of the intestinal barrier and regulating the immune system, but, when increased, affect immune reconstitution [184,185], were positively associated with anti-Dsg1 autoantibody titers [17]. Of interest, *Faecalibacterium*, known to exert anti-inflammatory actions through the production of butyrate [186], was also depleted in patients with autoimmune diseases (multiple sclerosis, Sjögren’s syndrome, systemic lupus erythematosus) [187] and positively associated with both anti-Dsg1 and anti-Dsg3 antibody titers in patients with active pemphigus [17]. Finally, research by Han et al. [164] explored differences in gut microbiota composition in different bullous diseases. Regarding PV, patients were characterized by an enrichment of Proteobacteria compared with healthy controls [164]. This is a signature of gut dysbiosis, given the generally low prevalence of this phylum within a balanced gut microbial community [188]. An increased abundance of Proteobacteria has, in fact, been associated with low-grade inflammatory conditions such as irritable bowel syndrome [189], obesity and metabolic disorders [190], and with severe intestinal inflammatory disorders including inflammatory bowel disease [191] and colorectal cancer [192]. Furthermore, consistent with Guo et al. [173], *Enterobacter* spp., which behaves as both facultative anaerobic Gram-negative natural commensals and opportunistic pathogens (behavior observed for *Enterobacter aerogenes*, *Enterobacter cloacae*, and *Enterobacter hormaechei*), were positively associated with Dsg3 autoantibodies, thus representing potential inducers of PV [148].

Overall, although the data provided so far are limited, a fair amount of evidence suggests that the composition of the intestinal microbiota of PV patients enriched in pathogenic and/or opportunistic bacteria (e.g., *Flavonifractor*, *Enterobacter*, *Escherichia coli*, *Bacteroides fragilis*) and with a lower relative abundance of probiotics (e.g., *Lachnospiracea_incertae_sedis*, *Coprococcus*, *Veillonella*), might be involved in the etiopathogenesis of the disease. However, the inconclusive results on associations between specific taxa and PV autoantibodies reflect the limitations of these studies, which are all based on a cross-sectional design and rely on too small numbers of subjects, and at the same time, they are often difficult to compare due to differences in sequencing methods and depths. Future multicenter research is desirable to increase statistical power together with experimental studies to confirm the role of microbial species in disease development and also to clarify all the underlying mechanisms involved in this relationship.

## 5. The Oral Microbiota and the Relationship with the Health-Disease Status

The oral cavity, a complex system of microbial habitats (buccal mucosa, teeth, gingival sulcus, hard and soft palate, tongue, and tonsils), hosts a multitude of bacteria, fungi, and viruses, among which bacteria represent the main components with over 700 species for a total of 150 genera and seven phyla (Bacteroidetes, Firmicutes, Actinobacteria, Fusobacteria, Proteobacteria, Spirochaetes, and TM7), making this microbial community the second largest after that of the gastrointestinal tract [193,194,195]. Unlike the gut microbiota, which can profoundly modify its composition based on changes in diet and local environment, the oral microbiota remains largely stable over time with no significant differences even between individuals, except for differences in relative abundance between taxa and at the strain level [193,194].

Growing evidence indicates that, in addition to covering a critical role in oral health by increasing the risk of oral diseases such as dental caries, periodontal disease, and oral cancer, the oral microbiota is also linked to a variety of extraoral conditions, including type 2 diabetes, obesity, inflammatory bowel disease, cardiovascular and pulmonary diseases, hepatitis, colon, esophageal, and pancreatic cancers, rheumatoid arthritis, low birth weight, Alzheimer’s disease, and Parkinson’s disease [37,193,194,196]. In fact, despite the still limited data, it has become clear that oral health, which can be profoundly affected by poor oral hygiene behaviors, can impact human health through an inflammatory response caused by a localized infection like gingivitis and mucosal inflammation or, alternatively, by the ectopic invasion of oral microorganisms (e.g., *Streptococcus mutans*, *Streptococcus sanguis*, *Fusobacterium nucleatum*, *Porphyromonas gingivalis*) into other organs [37,195,197]. Dysbiosis in the oral cavity creates an inflammatory microenvironment containing elevated levels of cytokines (e.g., IL-1, IL-2 IL-8, TNF-α, and prostaglandins), which can be released into the circulatory system and contribute to systemic inflammation [197].

Among chronic inflammatory cutaneous diseases, psoriasis is strongly associated with periodontal disease. In fact, psoriatic patients suffer from worse periodontal health (more severe gingival inflammation, alveolar bone loss, and fewer remaining teeth) compared with subjects without psoriasis [197,198]. A recent study also found that subjects with alterations in the oral microflora, i.e., reduced levels of *Prevotella* and higher relative abundance of *Corynebacterium*, are more likely to experience a psoriasis exacerbation, suggesting a direct contribution of the oral microbiota, rather than the severity of gingivitis, to the disease pathogenesis [196].

### The Association of the Oral Microbiota with Pemphigus Vulgaris

While the association between the oral microbiota and BP remains unexplored to date, two investigations have explored the oral microbiota profile in PV patients (Table 3). The first, which evaluated the composition of the microbiota in three different districts of patients affected by PV, highlighted how their oral mucosa was characterized by the greatest diversity, with Firmicutes as the most abundant phylum, followed, in decreasing proportions, by Fusobacteria, Bacteroidetes, Proteobacteria, and Actinobacteria [38]. Comparison with a selected reference control group revealed a significant decrease in the relative abundance of Bacteroidetes in the oral mucosa of PV patients, which could explain the typical intraoral halitosis resulting from the decomposition of sulfur-containing amino acids by anaerobic bacteria observed in these subjects [38,199]. The second study, aimed at assessing the composition of the oral microbiota in PV patients with oral lesions and healthy individuals, found a total of nine phyla in all cases and controls, and Firmicutes was confirmed as the most represented phylum in both groups, followed by Bacteroidetes, Proteobacteria, Actinobacteria, and Fusobacteria, although with a significant dominance of Firmicutes, Proteobacteria, and Fusobacteria in patients compared with healthy controls [200]. Despite the lack of substantial differences in the alpha diversity index, the significantly increased number of genera observed in patients might depend on insufficient oral hygiene due to the presence of painful lesions, which represent one of the early manifestations in 50% of PV subjects before manifesting as skin blisters [200,201]. On the other hand, the beta diversity analysis revealed a higher abundance of *Parvimonas micra* and *Fusobacterium nucleatum* in patients compared with controls [200]. *Parvimonas micra*, a gram-positive obligate anaerobe and a common constituent of the commensal flora in the gastrointestinal tract, has been identified as a major oral pathogen associated with endoperiodontal lesions and periodontitis, as well as among the most common species contributing to a variety of infections, systemic abscesses, and some malignancies [202,203,204,205]. *Fusobacterium nucleatum*, a gram-negative anaerobic opportunistic pathogen that promotes colonization and adherence to oral biofilms by almost all bacterial species, thus participating in the production of volatile sulfur compounds, and oral plaque formation, is also crucially involved in the development of periodontitis, extraoral infections and abscesses, colorectal cancer, and, with less evidence, oral squamous cell carcinoma [206,207,208,209]. The dominance of these two species, along with the depletion of *Streptococcus salivarius*, which appears to inhibit the growth of periodontal pathogens contributing to the balance of the oral microbiota, may explain the unpleasant odor of PV subjects [200,210].

While collectively, oral microbiota composition does not appear to be associated with PV, which also raises the question of whether dysbiosis status in the oral cavity is the cause or consequence of autoimmune bullous diseases, the higher relative abundance of oral Firmicutes in these patients suggests a possible role of this phylum in the PV pathogenesis. At the same time, the substantial reduction of Bacteroidetes and the abundance of anaerobic species associated with inflammatory diseases, including periodontitis, can account for the distinct malodor typical of PV patients.

## 6. The Skin Microbiota and the Relationship with the Health–Disease Status

Human skin, with a surface of approximately 1.8 m^2^, is the largest and the most exposed organ of the body, serving both as a physical barrier against foreign pathogens and as a niche capable of providing a milieu for a myriad of commensals, with a total of 1 million resident bacteria per cm^2^ [211,212]. Actinobacteria, Firmicutes, Proteobacteria, and Bacteroidetes are the four dominant bacterial phyla in the skin, with variations in microbial composition dictated by topography, whereby different regions characterized by specific pH, temperature, moisture, and sebum content influence microbial community structures [213,214]. Like the gut microbiota, the skin microflora represents a dynamic system, subject to two significant changes, the first one occurring after birth and the latter happening at the adolescence stage when sexual maturation promotes the proliferation of lipophilic bacteria, while other small changes are due to alterations in host biology and environmental exposures (skin care products, antibiotics) throughout life [211].

Despite the recent interest in the skin microbiota, it has been established to play a key role in maintaining healthy skin conditions through modulation of the immune system, inhibition of pathogenic bacteria growth, synthesis of vitamins and amino acids, and regulation of epidermal differentiation [211,214]. Epidermal keratinocytes produce AMPs (mostly represented by peptides such as β-defensin 2 and cathelicidin) that can be constitutively expressed or, alternatively, regulated by the complement C5a receptor, which also modulates the expression of pattern recognition receptors, proinflammatory mediators, and possibly the skin microbiota [215]. On the other hand, a disequilibrium in the composition of the microbial community, leading to epithelial barrier breakdown, increased pathogens colonization concomitant with depletion of beneficial commensals, and ultimately immune dysregulation and inflammatory response, has been associated with the onset of antigen-driven disorders including acne vulgaris (imbalance between the status of *Propionibacterium acnes* and *Staphylococcus epidermidis* [216]); psoriasis (increased proportions of Firmicutes, Bacteroidetes, *Streptococcus*, *Staphylococcus aureus* and reduction of Actinobacteria, Proteobacteria, Propionibacterium and *Staphylococcus epidermidis* [85,217,218]); atopic dermatitis (overgrowth of *Staphylococcus aureus* [219]); and rosacea (higher relative abundance of *Staphylococcus*, *Cutibacterium*, *Pseudomonas*, *Corynebacterium*, *Acinetobacter*, *Snodgrasella*, *Corynebacterium kroppenstedtii*, *Cutibacterium acnes* and *Staphylococcus epidermidis* [220,221,222]). Nonetheless, it is currently unclear whether inflammatory skin diseases are the result of disturbances in the skin microbiota or whether they themselves promote this change [214,223]. Importantly, in immunosuppressed hosts, certain species such as coagulase-negative *Staphylococcus*, *Corynebacterium*, *Malassezia*, *Cutibacterium acne*, and *Roseomonas mucosa* can cause systemic diseases when they penetrate deeper tissue, giving rise to bacteremia, chronic wounds, osteomyelitis, and/or surgical site soft tissue infections [224].

### The Association of the Skin Microbiota with Bullous Pemphigoid and Pemphigus Vulgaris

So far, the relationship between skin microbial communities and autoimmune blistering diseases has been investigated in three studies (Table 4). In the first, a comparison of the skin microbiota in BP patients and matched controls across multiple sites documented no significant differences in the alpha diversity, in agreement with most studies evaluating this index for the gut and oral microbiota (see previous sections) [5]. Conversely, beta diversity significantly differed between patients and controls in perilesional sites (areas adjacent to a fresh blister of erosion) as well as between perilesional and non-lesional sites (contralateral to the perilesional site) within patients, indicating that this parameter changed depending on the disease status and the associated cutaneous microbiota [5]. Perilesional sites in BP patients had a higher relative abundance of Firmicutes and *Staphylococcus* [5], which, in *Staphylococcus aureus*, is associated with atopic dermatitis that, like BP, shares various characteristics, including itch, increased levels of eosinophils, and elevated amounts of IgG autoantibodies [6,225,226,227,228]. Scaglione et al. [38] recruited patients with PV or BP and assessed the composition of the skin lesion microbiota, finding that Firmicutes was the most represented phylum in both patient groups, while the genus *Staphylococcus* was confirmed as the most abundant in the two groups. In contrast to the study by Miodovnik et al. [5], who reported a higher relative abundance of *Staphylococcus epidermis*, an opportunistic pathogen in the context of immunosuppression, the authors identified *Staphylococcus aureus* as the most abundant species in BP patients [38], consistent with previous data [229,230]. In addition to being implicated in the pathogenesis of atopic dermatitis and other minor skin infections such as folliculitis and impetigo, *Staphylococcus aureus*, a commensal species that is a major component of the skin microbiota, has also been associated with systemic lupus erythematosus with renal and skin involvement [231] and is the leading etiologic agent of osteomyelitis [232], septic arthritis [233], and sepsis [234]. Furthermore, *Staphylococcus aureus* can colonize chronic wounds typical of the genetic blistering disease epidermolysis bullosa [235]. Notably, in line with Miodovnik et al. [5], who identified Proteobacteria as the most abundant phylum in non-lesional sites of BP patients and healthy subjects, Scaglione and coauthors found a prevalence of Proteobacteria >5% in only one patient, supporting the low pathogenicity of this phylum [38]. In a recent multicenter study [236], substantial differences in the skin microbiota were detected between BP patients and age- and sex-matched controls, with a significant reduction in the alpha diversity in both perilesional and contralateral sites of patients, as also reported for epidermolysis bullosa acquisita [237], psoriasis [238], and atopic dermatitis [239]. The authors observed an inverse correlation of disease status with *Cutibacterium acnes*, one of the most abundant bacteria in the skin microbiota, which, although acting as an opportunistic pathogen leading to various inflammatory conditions both in the skin and many other internal organs, is generally considered a commensal species implicated in maintaining skin health through the production of SCFAs, which can limit pathogen colonization [236,240,241,242]. The relative abundance of *Staphylococcus aureus* was increased in both perilesional and contralateral lesions and in sites rarely affected by BP, consistent with the higher prevalence of this species in the nares and on the surface of unaffected skin of BP patients compared with matched controls observed by Messingham and coauthors [230] and the significant positive correlation with disease severity [236]. *Staphylococcus aureus* and *Staphylococcus hominis* also exhibited significantly negative correlations in perilesional and contralateral sites of patients but not in any matched controls, suggesting that *Staphylococcus aureus* is an important biomarker of BP, although it remains unclear whether PV patients were colonized by this species before developing the disease [215,221].

Overall, these findings indicate that the skin microbiota may play a key role in BP, although published cross-sectional studies do not allow us to exclude the observed differences in the composition of microbial communities (i.e., a higher relative abundance of Firmicutes in affected subjects) derive from the disease. In particular, the skin pathogen *Staphylococcus aureus* appears as a pivotal indicator of BP and is associated with the severity status of the disease, suggesting that modulation of the skin microbiota, even with early antibiotic treatment, could represent an effective therapeutic strategy for BP.

## 7. Conclusions and Future Challenges

In recent years, the role of microbiota, particularly in the human gut, has been increasingly investigated in both healthy subjects and diseases. Perturbations in the balance of microbial communities have been associated with a higher risk of developing a variety of pathological conditions through the production of metabolites that, acting as antigens, can stimulate intestinal epithelial cells, cause damage to the intestinal barrier, and trigger autoimmune responses and subsequent inflammation. BP and PV are the most common chronic autoimmune bullous diseases, and despite distinct physiopathology and clinical signs, their etiology, at least partly unknown, has been linked to an enrichment of pathogenic bacteria and a generally higher relative abundance of Firmicutes and to a depletion of probiotics/beneficial species, along with specific associations between certain bacterial taxa and disease autoantibodies and the involvement of microbial function alterations. Data gathered so far are suggestive of a potentially relevant role of changes in the microbiota composition and diversity in the development and progression of BP and PV, although with different levels of evidence depending on the body district (Table 5) composition.

While the relationship between the alpha and beta diversity indices in the skin and oral cavity and the prevalence of the two diseases is still poorly explored, growing evidence suggests that gut microbiota alterations in the beta diversity may contribute to BP risk, with certain microorganisms and pathways directly involved in the link between gut dysbiosis and disease activity. In contrast, the role of the gut microbiota in the etiopathogenesis of PV remains controversial, as only a few studies documented significant variations in the composition and diversity of microbial communities between patients and healthy controls. Overall, published studies are based on a cross-sectional design and a limited number of subjects, given the rarity of BP and PV. Additionally, most of the research was conducted in Asian countries, which represents a potential bias in the result interpretation due to the possible influence of lifestyle and dietary habits on the composition of the microbiota. Heterogeneity between studies owing to different sequencing methods (e.g., 16S rRNA gene sequencing, metagenomic analysis, and metabolome sequencing), populations, diet, age-related alterations in the gut microbiota, disease severity, presence of comorbidities, and pharmacological treatments makes comparison of results difficult and needs further validation in future studies. Multicenter studies enrolling a large number of subjects and possibly performed in different geographical areas are therefore warranted to verify the current data and the possibility of a causal relationship, also through the analysis of microbe-microbe interactions to determine which microorganisms and/or strains can be unequivocally associated with BP and PV development and which instead can be beneficial for their management, together with experimental studies aimed at identifying plausible biological mechanisms.

The knowledge gained so far, and future data will help to shed light on both a complete picture of the development and diagnostics of BP and PV, also by detecting bacterial markers and possible alternative treatment strategies for these two conditions. In particular, modulation of the intestinal microbiota by oral prebiotics (a group of nutrients, namely plant fiber, used to feed the gut commensals) and probiotics (live strains of selected microorganisms, mainly of *Lactobacillus* and *Bifidobacterium* genera, which, if administered in adequate amounts, confer a healthy balance to the host’s gut) have immunomodulatory, anti-inflammatory, and metabolic effects, thus acting as therapeutic agents [27]. Fecal microbiota transplantation (FMT), aimed at restoring the homeostasis conditions of the microbiota habitat and normal SCFA synthesis, has been successfully applied in the treatment of numerous disorders, such as Crohn’s disease, hepatic encephalitis, metabolic diseases, Parkinson’s disease, multiple sclerosis, and allergic and autoimmune diseases, and could therefore represent an additional treatment option in bullous diseases, also in light of the ongoing study of new methods to minimize the side effects of FMT and replenish only species with selected characteristics [27]. At the same time, the development of bacteriotherapy, which is based on the topical application of prebiotics and probiotics, the latter already in use to combat skin aging, could improve skin health by promoting the growth of beneficial species (e.g., *Staphylococcus epidermis*), eliminating pathogens (e.g., *Staphylococcus aureus*), and enhancing the skin’s natural defense barrier [211,212,214]. Therefore, the collected findings, in addition to providing new insights into the pathogenesis of bullous diseases, pave the way for the beginning of a novel era in their treatment through a holistic approach, including the use of multiple methods, such as FMT, oral and topical pre- and probiotics, and, possibly, the use of antibiotics against oral opportunistic infections occurring in PV as adjuvants to traditional therapies.

## Figures and Tables

**Table 1:** Summary of the main characteristics of studies exploring the role of the gut microbiota in the etiopathogenesis of bullous pemphigoid.

Study Design	Country— Study Period	Population	Main Findings	Limitations	References
Cross-sectional	Italy— January 2018–June 2018	8 BP patients: 5 females, mean age 70 ± 18 years	Firmicutes relative abundance: p50 (%, min–max): 47.7 (38.8–65.3). Bacteroidetes relative abundance: p50 (%, min–max): 43.8 (33.0–50.9). Proteobacteria relative abundance: p50 (%, min–max): 7.8 (5.2–12.7).	Small sample size.	[38]
Cross-sectional	Germany (N = 14; Finland (N = 3); Bulgaria (N = 3)— June 2014–July 2020	18 BP patients: 46 females, mean age 40.26 (63–98) years 66 healthy controls: 46 females, mean age 80.64 (62–100) years. Two subgroups: 54 pairs, first diagnosis (BPF and CLF), and 11 pairs, relapse (BPR and CLR)	Significant decrease and a lower trend in the Chao and Shannon indexes, respectively, in BP patients. Significant decrease in Chao index in BPR compared with the CLR group. Significant difference in Bray–Curtis dissimilarity between BP patients and their healthy controls and between the first diagnosis cases and their controls but not between relapsed cases and their matched controls. Microbial composition affected by study center, disease status, and age. At the genus level, *Flavonifractor* significantly and primarily enriched in BPR cases, while *Faecalibacterium* reduced in both subgroups of patients. At the species level, *Ruthenibacterium lactatiformans*, *Anaerotruncus colihominis*, and *Eubacterium callanderi* significantly increased, and *Prevotella copri*, *Faecalibacterium prausnitzii*, and *Faecalibacterium* sp. *I417* significantly decreased in BP patients if compared with their controls. *Ruthenibacterium lactatiformans*, *Anaerotruncus colihominis*, *Bacteroides eggerthii,* and *Bifidobacterium dentium* more enriched, and *Sutterella wadsworthensis* reduced in the BPF group compared with the CLF group. Significant increase in six bacterial species, including *Flavonifractor plautii*, and significant decline of three species, including *Alistipes shahii*, in BPR cases compared with their matched controls. Twelve gut microbial pathways significantly affected in BP patients. 30 species and 49 pathways significantly associated with BPDAI score.	Lack of longitudinal and metabolome data. Small sample size. Controls included subjects with basal cell carcinoma or squamous cell carcinoma.	[10]
Cross-sectional	China	24 BP-O patients: 7 females, mean age 69.75 ± 10.28 years. 24 BP-R patients: 11 females, mean age 69.92 ± 13.50 years 26 healthy controls: 14 females, mean age 63.58 ± 9.15 tears	No significant differences in ACE index between the three groups, except for a higher score in the BP-O group compared with healthy controls and a lower score in BP-R patients than the two other groups. No significant differences in Bray–Curtis dissimilarity between the three groups. At the family level, the BP-O group showed an increased proportion of Bacteroidaceae, Ruminococcaceae, and Enterobacteriaceae and a lower proportion of Lachnospiraceae, Prevotellaceae, and Veillonellaceae compared with the healthy controls. In the BP-R group, the proportion of Lachnospiraceae and Veillonellaceae increased and that of Ruminococcaceae, Bacteroidaceae, Prevotellaceae and Enterobacteriaceae decreased compared with the control group. At the ASV level, similarity between BP-R and control groups and significant differences in enriched and depleted ASVs between BP-O and the other two groups. Significantly higher relative abundance of *Prevotella copri* and significant depletion of *Vellonella dispar* and *Bacteroides ovatus* in the BP-O group compared with other groups. *Barnesiella intestinihominis* and *Veillonella dispar* inversely correlated with anti- BP180, while *Ruminococcus albus*, *Vescimonas coprocola*, *Sporobacter termitidis*, *Alistipes shahii*, and *Bifidobacterium adolescentis* positively correlated with anti-BP180. *Prevotella copri*, *Roseburia intestinalis*, and *Sutterella wadsworthensis* inversely correlated with EOS%, while *Blautia hominis* positively correlated with EOS%. In BP-O patients, enrichment of *Ruminococcaceae* spp., *Clostridium XIVb*, *Coprococcus*, *Oscillibacter*, and *Escherichia Shigella*. In BP-R patients, enrichment of *Lachnospiracea incertae sediswere* and *Sutterella*.	Lack of longitudinal data. Small sample size. Single-center study. No standardized criteria for patients’ complications. The effect of the treatment on the gut microbiota not evaluated. Lack of assessment of the relationship between gut microbiota composition and anti-BP230.	[39]
Prospective	China— October 2016–March 2022	38 BP patients: 12 females, mean age 67.8 ± 11.4 years 38 healthy controls: 22 females, mean age 65.5 ± 10.3 years	No significant difference in the Shannon index between BP patients and controls. Bray–Curtis dissimilarity significantly different between cases and controls. At the phylum level, enrichment of Proteobacteria and Actinobacteria in BP patients. At the genus level, BP patients show an increase in Bacteroides and *Prevotella* and decrease in *Escherichia-Shigella* and *Faecalibacterium*. Significant differences between cases and controls in the microbial composition of Bacteroidetes and Firmicutes phyla. *Faecalibacterium* negatively correlated with anti-BP180. Alterations in 14 functional pathways significantly increased in BP patients.	Small sample size. Single-center study. Lack of metagenomic analysis.	[164]

Abbreviations: ASV: amplicon sequence variant; BP: bullous pemphigoid; BPDAI: Bullous Pemphigoid Disease Area Index; BPF: bullous pemphigoid first diagnosis; BP-O: BP onset; BP-R: BP under remission stage; BPR: bullous pemphigoid relapse; CLF: matched controls with bullous pemphigoid first diagnosis; CLR: matched controls with bullous pemphigoid relapse; EOS%: percentage of eosinophils.

**Table 2:** Summary of the main characteristics of studies exploring the role of the gut microbiota in the etiopathogenesis of pemphigus vulgaris.

Study Design	Country— Study Period	Population	Main Findings	Limitations	References
Cross-sectional	Italy— January 2018–June 2018	12 PV patients: 6 females, mean age 55 ± 14 years	Firmicutes relative abundance: p50 (%, min–max): 43.3 (31.8–75.0). Bacteroidetes relative abundance: p50 (%, min–max): 50.9 (20.5–66.6). Proteobacteria relative abundance: p50 (%, min–max): 10.3 (5.7–21.4).	Small sample size.	[38]
Cross-sectional	China— January 2017–May 2020	18 PV patients: 9 females, mean age 45.78 ± 13.45 years 14 healthy controls: 5 females, mean age 44.57 ± 14.72 years	No significant differences in the Shannon and Simpson diversity indexes between cases and controls. Ten taxa significantly different between the two groups. At the family level, higher abundance of Carnobacteriaceae, Enterobacteriaceae, and Burkholderiales and reduced levels of Enterobacteriales in PV. At the genus level, PV patients have decreased levels of *Lachnospiracea_incertae_sedis* and *Coprococcus* and increased levels of *Granulicatella* and *Flavonifractor*. Significantly higher concentration of six cytokines (IL-1β, IL-2R, IL-7, IL-8, C5a, YKL-40) out of 21 overall assessed in PV group than in the control group. Plasma IL-5, IL-6, IL-17A, and IL-21 show an increasing trend in PV patients. Significant positive correlation between *Flavonifractor* and plasma levels of C5a, IL-1β, IL-6, IL-7, IL-8, and IL-21. Significant inverse correlation of *Lachnospiracea_incertae_sedis* and *Coprococcus* with plasma IL-17A concentration.	Lack of longitudinal data. Small sample size. Single-center study. Half of PV patients under treatment with systemic corticosteroids. Lack of possibility to infer a causal relationship due to the study design.	[153]
Cross-sectional	China	43 PV patients: 15 females, mean age 51.89 ± 15.61 years 26 healthy controls: 11 females, mean age 52.92 ± 15.21 years	No significant differences in the Richness, Chao, and Shannon indexes between cases and controls. Simpson index significantly higher in healthy controls than in PV patients. High level of dissimilarity between the two groups based on Bray–Curtis analysis. Decrease in the relative abundance of Firmicutes and increase in that of Proteobacteria and Verrucomicrobia in PV patients. At the genus level, higher proportions of *Bacteroides*, *Escherichia*, *Akkermansia*, and *Klebsiella* and lower proportions of *Faecalibacterium* and *Roseburia* in the case group. Opportunistic pathogens (unclassified *Klebsiella*, *Bacteroides fragilis*, and *Bacteroides thetaiotaomicron*) positively correlated with PDAI and anti-Dsg1 and anti-Dsg3 antibody levels. 215 significant associations between enriched bacterial species and metabolites.	Lack of longitudinal data. Single-center study. Small disease cohort. Results data-driven. Lack of possibility to infer a causal relationship due to the study design.	[173]
Cross-sectional	China— November 2017–April 2019	60 PV patients: 33 females, mean age 47.38 ± 12.82 years 19 matched healthy family members: 9 females, mean age 41.00 ± 14.81 years 100 fecal samples (60 treatment-naïve, 21 matched post-treatment, and 19 controls)	No significant differences in alpha diversity. Significantly high degree of dissimilarity between the two groups (beta-diversity). Three enterotypes—E1, E2, E3—identified in the two groups: E2 (Escherichia predominant) and E3 (Bacteroides predominant) significantly enriched in PV and healthy controls, respectively. At the phylum level, Actinobacteria predominant in PV patients, while Bacteroidetes predominant in healthy controls. At the species level, PV patients have a significant decrease in *Bacteroidesovatus*, *Bacteroides uniformis*, *Eubacterium rectale*, *Eubacterium ventriosum*, *Roseburia intestinalis*, and *Roseburia inulinivorans* and significant enrichment in *Escherichia coli*. *Lachnospiraceae bacterium 5.1.57FAA* abundance significantly and positively correlated with anti-Dsg3 antibodies and PDAI scores. *Eubacterium ventriosum* strongly and positively correlated with the ΔPDAI (an index that reflects the response to glucocorticoid treatment). Higher abundance of *Escherichia coli* in responders than in non-responders to therapy. No significant variations in alpha and beta diversity after glucocorticoid treatment; however, after one month of therapy, there was a decrease in the relative abundance of Escherichia coli and an increase in the probiotic abundance. PTS pathway, the most represented in PV patients, showing the strongest correlation with Escherichia coli. Fatty acid biosynthesis enriched in healthy controls and had the highest correlation with *Bacteroides ovatus*.	Lack of longitudinal data. Single-center study. Small sample size. Lack of a control group for other autoimmune diseases. The mechanisms by which Escherichia coli participates in the development of PV not investigated. Lack of possibility to infer a causal relationship due to the study design.	[117]
Cross-sectional	China— November 2016–May 2022	20 patients with AP (15 of whom with PV): 11 females, mean age 52.80 ± 16.79 years 11 patients with PR (7 of whom with PV): 6 females, mean age 60.36 ± 12.31 years 47 healthy controls (most of them spouses of the patients): 29 females, mean age 62.62 ± 11.45 years	No significant differences in the indexes of alpha diversity, but a progressive decrease in ACE and Chao indexes from healthy controls to the PR group and then to the AP group and a slight decrease of Shannon and Simpson indexes in the healthy control group compared with the other groups. No significant differences in beta diversity. Firmicutes and Bacteroidetes dominant phyla in all three groups, with a decreasing, albeit not significant, trend of the F/B ratio in AP patients. At the family level, the highest relative abundance of Lachnospiraceae in PR and the lowest relative abundance of Veillonellaceae in AP. Prevotalleceae abundance progressively increased in the AP, healthy control, and PR groups without significant differences. At the genus level, *Blautia* abundance is significantly higher in the AP than in the PR group, while that of *Prevotella* shows a not significant increase across AP, healthy control, and PR groups.	Lack of longitudinal data. Small sample size. Single-center study. Lack of adjustment for dietary habits between cases and controls. Some patients under systemic corticosteroid treatment. Significant age differences between the three groups. Lack of possibility to infer a causal relationship due to the study design.	[17]
Cross-sectional	China— October 2016–March 2022	19 PV patients: 11 females, mean age 59.9 ± 15.0 years 38 healthy controls: 22 females, mean age 65.5 ± 10.3 years	No significant difference in the Shannon index and Bray–Curtis dissimilarity between PV patients and controls. At the phylum level, enrichment of Proteobacteria and Actinobacteria in PV patients. At the genus level, increase in *Bacteroides* and *Faecalibacterium* and a decrease in *Escherichia-Shigella* and *Prevotella* among cases. At the species level, enrichment in *Intestinibacter bartletti* in PV patients and *Blautia wexlerae* and *Bifidobacterium catenulatum* in controls. Significant differences between cases and controls in the microbial composition of Bacteroidetes and Proteobacteria phyla. *Enterobacter* positively correlated with anti-Dsg3.	Lack of longitudinal data. Small sample size. Single-center study. Lack of metagenomic analysis.	[164]

Abbreviations: AP: active pemphigus; Dsg: desmoglein; IL: interleukin; PDAI: Pemphigus Disease Area Index; PR: pemphigus remission; PTS: phosphotransferase system; PV: pemphigus vulgaris.

**Table 3:** Summary of the main characteristics of studies exploring the role of the oral microbiota in the etiopathogenesis of pemphigus vulgaris.

Study Design	Country— Study Period	Population	Main Findings	Limitations	References
Cross-sectional	Italy— January 2018–June 2018	12 PV patients: 6 females, mean age 55 ± 14 years	Firmicutes relative abundance: p50 (%, min–max): 45.5 (27.1–72.6) in PV patients vs. 39.6 (32.3–73.4) in healthy controls. Fusobacteria relative abundance: p50 (%, min–max): 28.0 (10.4–41.6) in PV patients vs. 8.5 (1.9–13.2) in healthy controls. Bacteroidetes relative abundance: p50 (%, min–max): 7.2 (5.7–12.6) in PV patients vs. 8.5 (1.9–13.2) in healthy controls. Proteobacteria relative abundance: p50 (%, min–max): 15.2 (5.1–23.9) in PV patients vs. 13.3 (10.5–42.6) in healthy controls. Actinobacteria relative abundance: p50 (%, min–max): 5.5 (2.8–27.0) in PV patients vs. 2.4 (1.4–5.3) in healthy controls.	Small sample size.	[38]
Cross-sectional	Greece— January 2016–December 2018	15 PV patients: 9 females 15 healthy controls	At the phylum level, significant differences in the relative abundance of Firmicutes (61.27% in patients vs. 55.88% in controls), Proteobacteria (12.33% in patients vs. 9.17% in controls), Fusobacteria (4.09% vs. 3.39%). At the family level, significant differences in the relative abundance of *Bacillales incertae* sedis (5.98% in patients vs. 1.41% in controls) and Fusobacteriaceae (3.91% in patients vs. 2.56% in controls). At the genus level, significant differences in the relative abundance of *Streptococcus* (34.37% in patients vs. 33.30% in controls), *Fusobacterium* (4.51% in patients vs. 4.13% in controls), and *Gemella* (7.13% in patients vs. 5.80% in controls). Alpha diversity: no significant differences in Shannon, Simpson, and Fischer indexes. Eleven taxa significantly increased in abundance in controls and 30 taxa in patients. Patients showing the highest mean of the phylum Firmicutes, families Clostridiales Family XI Incertae Sedis and Carnobacteriaceae, genera *Actibacillus*, *Aggregatibacter*, *Selenomonas*, *Prevotella* with species *P. maculosa*, *nigrescens*, *oris*, and other sp., and *Streptococcus* with species *S. intermedius*, *mitis*, *sanguinis,* and *thermophilus*. Bray–Curtis dissimilarity significantly different between patients and controls, with Firmicutes and Fusobacteria significantly enriched in cases compared with healthy subjects. Smoker patients with a high abundance of *Rothia mucilaginosa*, *Streptococcus salivarius*, *Haemophilus parainfluenzae*, *Granulicatella adiacens*, and *Streptococcus pseudopneumoniae*. 11 species only detected in smokers. Eleven significantly discriminative taxa between smokers and non-smoker patients, with Firmicutes having the highest proportion in smokers and Proteobacteria in non-smokers.	Lack of longitudinal data. Small sample size. Single-center study.	[200]

Abbreviations: PV: pemphigus vulgaris.

**Table 4:** Summary of the main characteristics of studies exploring the role of the skin microbiota in the etiopathogenesis of bullous pemphigoid and pemphigus vulgaris.

Study Design	Country— Study Period	Population	Main Findings	Limitations	References
Cross-sectional	Germany— August 2014–January 2015	12 BP patients: 9 females, mean age 79.8 ± 9.9 years 12 healthy controls: 7 females, mean age 81.7 ± 7.5 years	Shannon, Simpson, and Chao1 indexes not significantly different between perilesional and non-lesional sites in BP patients. Bray–Curtis dissimilarity index significantly different by sample location, blistering disease status, group affiliation, and between perilesional and non-lesional sites within patients. Significant decrease in Actinobacteria abundance in back, elbow, and perilesional samples from patients and in Proteobacteria in perilesional sites. Significant enrichment of Firmicutes and the genus *Staphylococcus* in patient perilesional sites compared with control sites matched controls. At the species level, relative abundance of *Staphylococcus epidermis* significantly different between patients and controls on perilesional sites. Actinobacteria abundance significantly different between perilesional and non-lesional sites in patients. Proteobacteria as the most abundant phylum in control and patient non-lesional sites; Actinobacteria and Firmicutes as the second most abundant phylum in control and patient non-lesional sites, respectively. Higher relative abundance of Firmicutes in perilesional sites in patients. Four-fold increase in the Firmicutes/Proteobacteria ratio in BP compared with control subjects.	Lack of longitudinal data. Small sample size. Single-center study.	[5]
Cross-sectional	Italy— January 2018–June 2018	12 PV patients: 6 females, mean age 55 ± 14 years 8 BP patients: 5 females, mean age 70 ± 18 years	Firmicutes relative abundance: p50 (%, min–max): 82.4 (82.1–83.2) in PV patients vs. 99.3 (55.7–99.9) in BP patients. Actinobacteria relative abundance: p50 (%, min–max): 17.4 (15.8–17.5) in PV patients vs. 30.7 in BP patients (only one subject). Proteobacteria relative abundance: p50 (%, min–max): 13.4 in BP patients (only one subject). *Staphylococcus* and *Corynebacterium* species enriched in BP patients. Overall increased diversity of bacterial species in PV patients Shannon index significantly different between the two groups.	Small sample size. Single-center study.	[38]
Cross-sectional	Germany; France; Bulgaria; Greece; Finland— October 2015–September 2019	228 BP patients: 113 females, mean age 80 ± 8.95 years 190 healthy controls: 86 females, mean age 80 ± 8.51 years	Shannon and Chao1 indexes not significantly different at sites rarely affected by BP. Control corresponding sites showing higher bacterial diversity than patient contralateral sites. Contralateral sites showing higher bacterial diversity than perilesional sites in patients. Shannon index significantly correlated with study center, disease status, and sex. Chao1 index significantly correlated with the study center and disease status. Disease status associated with a decrease in Shannon index in perilesional and contralateral lesions in patients and with a decrease in Chao1 index, even after adjustment for covariates. BPDAI not significantly associated with alpha diversity indexes at perilesional and contralateral skin sites. Bray–Curtis dissimilarity index significantly correlated with disease status. Disease status, blistering status, and study center accounting for a portion of the variance in the beta-diversity. *Cutibacterium acnes* abundance significantly correlated with study center, blistering status, and sex and higher relative abundance at control corresponding sites than at perilesional sites in patients. *Staphylococcus hominis* abundance significantly correlated with disease status and body site and significantly inversely correlated with BPDAI at patient contralateral sites but not perilesional sites. *Staphylococcus aureus* abundance significantly correlated with disease status at rarely affected sites. Decrease in *Staphylococcus aureus* abundance in control corresponding sites compared with an increase in patient perilesional sites. *Staphylococcus aureus* positively correlated with BPDAI at perilesional and contralateral sites but not correlated with age at any patient sites. *Staphylococcus hominis* and *Staphylococcus aureus* significantly negatively correlated with patient perilesional and contralateral sites but not with any matched control sites. *Staphylococcus aureus* significantly negatively correlated with *Cutibacterium acnes* at all patient sites but not in matched controls.	Lack of longitudinal data.	[236]

Abbreviations: BP: bullous pemphigoid; BPDAI: Bullous Pemphigoid Disease Area Index; PV: pemphigus vulgaris.

**Table 5:** Summary table of the qualitative level of evidence on the association between variations in the microbiota at different body districts and risk of development of bullous pemphigoid and pemphigus vulgaris.

Autoimmune Skin Disease	Body District	Changes in the Microbiota	Level of Evidence
Bullous pemphigoid	Gut	Alpha diversity—no significant variations; beta-diversity significantly different in cases vs. controls	Moderate
	Oral cavity	No studies	-
	Skin	Alpha diversity significantly correlated with the disease status in one study; beta-diversity significantly different in cases vs. controls in one study	Low
Pemphigus vulgaris	Gut	Alpha diversity generally not significantly different between cases and controls; high degree of changes in the beta diversity in some studies	Low
	Oral cavity	Alpha diversity—no significant variations; beta-diversity significantly different in cases vs. in one study	Low
	Skin	Significant variations in the alpha diversity in one study	Low

## Abbreviations

The following abbreviations are used in this manuscript:

AMP	Antimicrobial peptide
BA	Bile acid
BP	Bullous pemphigoid
Dsg	Desmoglein
F/B	Firmicutes/Bacteroidetes ratio
FMT	Fecal microbiota transplantation
GABA	γ-aminobutyric acid
GAD	Glutamate decarboxylase
GALT	Gut-associated lymphoid tissues
GPCR	G protein-coupled receptor
H_2_S	Hydrogen sulfide
IEC	Intestinal epithelium cells
IFN-γ	Interferon gamma
Ig	Immunoglobulin
IL	Interleukin
LCA	Lithocholic acid
LPS	Lipopolysaccharide
NF-κB	Nuclear factor kappa-light-chain-enhancer of activated B cells
NO	Nitric oxide
NOD	Nucleotide-binding oligomerization domain
PAMP	Pathogen-associated molecular pattern
rRNA	ribosomal RNA
SCFAs	Short-chain fatty acids
Th	T helper
TLR	Toll-like receptor
TMAO	Trimethylamine-N-oxide
TNF-α	Tumor necrosis factor-alpha
Treg	Regulatory T cells

## References

[1] Kridin K. (2018). Pemphigus group: Overview, epidemiology, mortality, and comorbidities. Immunol. Res..

[2] Di Lernia V., Casanova D.M., Goldust M., Ricci C. (2020). Pemphigus Vulgaris and Bullous Pemphigoid: Update on Diagnosis and Treatment. Dermatol. Pract. Concept..

[3] Egami S., Yamagami J., Amagai M. (2020). Autoimmune bullous skin diseases, pemphigus and pemphigoid. J. Allergy Clin. Immunol..

[4] Lo Schiavo A., Ruocco E., Brancaccio G., Caccavale S., Ruocco V., Wolf R. (2013). Bullous pemphigoid: Etiology, pathogenesis, and inducing factors: Facts and controversies. Clin. Dermatol..

[5] Miodovnik M., Künstner A., Langan E.A., Zillikens D., Gläser R., Sprecher E., Baines J.F., Schmidt E., Ibrahim S.M. (2017). A distinct cutaneous microbiota profile in autoimmune bullous disease patients. Exp. Dermatol..

[6] Lin L., Hwang B.J., Culton D.A., Li N., Burette S., Koller B.H., Messingham K.A., Fairley J.A., Lee J.J., Hall R.P. (2018). Eosinophils Mediate Tissue Injury in the Autoimmune Skin Disease Bullous Pemphigoid. J. Investig. Dermatol..

[7] Nätynki A., Tuusa J., Hervonen K., Kaukinen K., Lindgren O., Huilaja L., Kokkonen N., Salmi T., Tasanen K. (2020). Autoantibodies Against the Immunodominant Bullous Pemphigoid Epitopes Are Rare in Patients With Dermatitis Herpetiformis and Coeliac Disease. Front. Immunol..

[8] Khalid S.N., Khan Z.A., Ali M.H., Almas T., Khedro T., Raj Nagarajan V. (2021). A blistering new era for bullous pemphigoid: A scoping review of current therapies, ongoing clinical trials, and future directions. Ann. Med. Surg..

[9] Persson M.S.M., Begum N., Grainge M.J., Harman K.E., Grindlay D., Gran S. (2022). The global incidence of bullous pemphigoid: A systematic review and meta-analysis. Br. J. Dermatol..

[10] Liu X., van Beek N., Cepic A., Andreani N.A., Chung C.J., Hermes B.M., Yilmaz K., Benoit S., Drenovska K., Gerdes S. (2023). The gut microbiome in bullous pemphigoid: Implications of the gut-skin axis for disease susceptibility. Front. Immunol..

[11] Cozzani E., Marzano A.V., Caproni M., Feliciani C., Calzavara-Pinton P., Cutaneous Immunology Group of SIDeMaST (2018). Bullous pemphigoid: Italian guidelines adapted from the EDF/EADV guidelines. Ital. Dermatol. Venereol..

[12] Persson M.S.M., Harman K.E., Vinogradova Y., Langan S.M., Hippisley-Cox J., Thomas K.S., Gran S. (2021). Incidence, prevalence and mortality of bullous pemphigoid in England 1998–2017: A population-based cohort study. Br. J. Dermatol..

[13] Borradori L., Van Beek N., Feliciani C., Tedbirt B., Antiga E., Bergman R., Böckle B.C., Caproni M., Caux F., Chandran N.S. (2022). Updated S2 K guidelines for the management of bullous pemphigoid initiated by the European Academy of Dermatology and Venereology (EADV). J. Eur. Acad. Dermatol. Venereol..

[14] Porro A.M., Seque C.A., Ferreira M.C.C., Enokihara M.M.S.E.S. (2019). Pemphigus vulgaris. An. Bras. Dermatol..

[15] Kridin K., Schmidt E. (2021). Epidemiology of Pemphigus. JID Innov..

[16] Quintarelli L., Coi A., Maglie R., Corrà A., Mariotti E.B., Aimo C., Ruffo di Calabria V., Verdelli A., Bianchi B., Del Bianco E. (2022). Clinical Patterns, Survival, Comorbidities, and Treatment Regimens in 149 Patients With Pemphigus in Tuscany (Italy): A 12-Year Hospital-Based Study. Front. Immunol..

[17] Li S.Z., Wu Q.Y., Fan Y., Guo F., Hu X.M., Zuo Y.G. (2024). Gut Microbiome Dysbiosis in Patients with Pemphigus and Correlation with Pathogenic Autoantibodies. Biomolecules.

[18] Langan S.M., Smeeth L., Hubbard R., Fleming K.M., Smith C.J., West J. (2008). Bullous pemphigoid and pemphigus vulgaris--incidence and mortality in the UK: Population based cohort study. BMJ.

[19] Zhao L., Chen Y., Wang M. (2023). The Global Incidence Rate of Pemphigus Vulgaris: A Systematic Review and Meta-Analysis. Dermatology.

[20] Didona D., Maglie R., Eming R., Hertl M. (2019). Pemphigus: Current and Future Therapeutic Strategies. Front. Immunol..

[21] Gallo R.L. (2017). Human Skin Is the Largest Epithelial Surface for Interaction with Microbes. J. Investig. Dermatol..

[22] Šuler Baglama Š., Trčko K. (2022). Skin and gut microbiota dysbiosis in autoimmune and inflammatory skin diseases. Acta Dermatovenerol. Alp. Pannonica Adriat..

[23] Gao T., Wang X., Li Y., Ren F. (2023). The Role of Probiotics in Skin Health and Related Gut-Skin Axis: A Review. Nutrients.

[24] Helander H.F., Fändriks L. (2014). Surface area of the digestive tract—Revisited. Scand. J. Gastroenterol..

[25] De Pessemier B., Grine L., Debaere M., Maes A., Paetzold B., Callewaert C. (2021). Gut-Skin Axis: Current Knowledge of the Interrelationship between Microbial Dysbiosis and Skin Conditions. Microorganisms.

[26] Hou Y., Li J., Ying S. (2023). Tryptophan Metabolism and Gut Microbiota: A Novel Regulatory Axis Integrating the Microbiome, Immunity, and Cancer. Metabolites.

[27] Senchukova M.A. (2023). Microbiota of the gastrointestinal tract: Friend or foe?. World J. Gastroenterol..

[28] Chai J., Deng F., Li Y., Wei X., Zhao J. (2024). Editorial: The gut-skin axis: Interaction of gut microbiome and skin diseases. Front. Microbiol..

[29] Belizário J.E., Faintuch J. (2018). Microbiome and Gut Dysbiosis. Exp. Suppl..

[30] Mahmud M.R., Akter S., Tamanna S.K., Mazumder L., Esti I.Z., Banerjee S., Akter S., Hasan M.R., Acharjee M., Hossain M.S. (2022). Impact of gut microbiome on skin health: Gut-skin axis observed through the lenses of therapeutics and skin diseases. Gut Microbes..

[31] Gorini F., Tonacci A. (2024). Vitamin D: An Essential Nutrient in the Dual Relationship between Autoimmune Thyroid Diseases and Celiac Disease-A Comprehensive Review. Nutrients.

[32] Mohammad S., Karim M.R., Iqbal S., Lee J.H., Mathiyalagan R., Kim Y.J., Yang D.U., Yang D.C. (2024). Atopic dermatitis: Pathophysiology, microbiota, and metabolome—A comprehensive review. Microbiol. Res..

[33] Peterle L., Sanfilippo S., Tonacci A., Li Pomi F., Borgia F., Gangemi S. (2023). Common pathogenetic traits of atopic dermatitis and autism spectrum disorders, potential connections and treatments: Trivial Th2 inflammation or much more?. Front. Immunol..

[34] Mann E.R., Lam Y.K., Uhlig H.H. (2024). Short-chain fatty acids: Linking diet, the microbiome and immunity. Nat. Rev. Immunol..

[35] Yoo J.Y., Groer M., Dutra S.V.O., Sarkar A., McSkimming D.I. (2020). Gut Microbiota and Immune System Interactions. Microorganisms.

[36] Hou K., Wu Z.X., Chen X.Y., Wang J.Q., Zhang D., Xiao C., Zhu D., Koya J.B., Wei L., Li J. (2022). Microbiota in health and diseases. Signal Transduct. Target. Ther..

[37] Peng X., Cheng L., You Y., Tang C., Ren B., Li Y., Xu X., Zhou X. (2022). Oral microbiota in human systematic diseases. Int. J. Oral. Sci..

[38] Scaglione G.L., Fania L., De Paolis E., De Bonis M., Mazzanti C., Di Zenzo G., Lechiancole S., Messinese S., Capoluongo E. (2020). Evaluation of cutaneous, oral and intestinal microbiota in patients affected by pemphigus and bullous pemphigoid: A pilot study. Exp. Mol. Pathol..

[39] Hu X., Wu Q., Fan Y., Guo F., Li S., Zhang S., Zuo Y.G. (2023). Identification of gut microbiota dysbiosis in bullous pemphigoid under different disease activity. Exp. Dermatol..

[40] Syromyatnikov M., Nesterova E., Gladkikh M., Smirnova Y., Gryaznova M., Popov V. (2022). Characteristics of the Gut Bacterial Composition in People of Different Nationalities and Religions. Microorganisms.

[41] Rinninella E., Tohumcu E., Raoul P., Fiorani M., Cintoni M., Mele M.C., Cammarota G., Gasbarrini A., Ianiro G. (2023). The role of diet in shaping human gut microbiota. Best. Pract. Res. Clin. Gastroenterol..

[42] Ames N.J., Ranucci A., Moriyama B., Wallen G.R. (2017). The Human Microbiome and Understanding the 16S rRNA Gene in Translational Nursing Science. Nurs. Res..

[43] Kho Z.Y., Lal S.K. (2018). The Human Gut Microbiome—A Potential Controller of Wellness and Disease. Front. Microbiol..

[44] Han D., Gao P., Li R., Tan P., Xie J., Zhang R., Li J. (2020). Multicenter assessment of microbial community profiling using 16S rRNA gene sequencing and shotgun metagenomic sequencing. J. Adv. Res..

[45] Kameoka S., Motooka D., Watanabe S., Kubo R., Jung N., Midorikawa Y., Shinozaki N.O., Sawai Y., Takeda A.K., Nakamura S. (2021). Benchmark of 16S rRNA gene amplicon sequencing using Japanese gut microbiome data from the V1-V2 and V3-V4 primer sets. BMC Genomics..

[46] Durazzi F., Sala C., Castellani G., Manfreda G., Remondini D., De Cesare A. (2021). Comparison between 16S rRNA and shotgun sequencing data for the taxonomic characterization of the gut microbiota. Sci. Rep..

[47] Zhang W., Fan X., Shi H., Li J., Zhang M., Zhao J., Su X. (2023). Comprehensive Assessment of 16S rRNA Gene Amplicon Sequencing for Microbiome Profiling across Multiple Habitats. Microbiol. Spectr..

[48] Rinninella E., Raoul P., Cintoni M., Franceschi F., Miggiano G.A.D., Gasbarrini A., Mele M.C. (2019). What is the Healthy Gut Microbiota Composition? A Changing Ecosystem across Age, Environment, Diet, and Diseases. Microorganisms.

[49] Pedroza Matute S., Iyavoo S. (2023). Exploring the gut microbiota: Lifestyle choices, disease associations, and personal genomics. Front. Nutr..

[50] Conlon M.A., Bird A.R. (2014). The impact of diet and lifestyle on gut microbiota and human health. Nutrients.

[51] Jiao Y., Wu L., Huntington N.D., Zhang X. (2020). Crosstalk Between Gut Microbiota and Innate Immunity and Its Implication in Autoimmune Diseases. Front. Immunol..

[52] Fusco W., Lorenzo M.B., Cintoni M., Porcari S., Rinninella E., Kaitsas F., Lener E., Mele M.C., Gasbarrini A., Collado M.C. (2023). Short-Chain Fatty-Acid-Producing Bacteria: Key Components of the Human Gut Microbiota. Nutrients.

[53] Gieryńska M., Szulc-Dąbrowska L., Struzik J., Mielcarska M.B., Gregorczyk-Zboroch K.P. (2022). Integrity of the Intestinal Barrier: The Involvement of Epithelial Cells and Microbiota-A Mutual Relationship. Animals.

[54] Paradis T., Bègue H., Basmaciyan L., Dalle F., Bon F. (2021). Tight Junctions as a Key for Pathogens Invasion in Intestinal Epithelial Cells. Int. J. Mol. Sci..

[55] Schoenborn A.A., von Furstenberg R.J., Valsaraj S., Hussain F.S., Stein M., Shanahan M.T., Henning S.J., Gulati A.S. (2019). The enteric microbiota regulates jejunal Paneth cell number and function without impacting intestinal stem cells. Gut Microbes..

[56] Paone P., Cani P.D. (2020). Mucus barrier, mucins and gut microbiota: The expected slimy partners?. Gut.

[57] Zhou A., Yuan Y., Yang M., Huang Y., Li X., Li S., Yang S., Tang B. (2022). Crosstalk Between the Gut Microbiota and Epithelial Cells Under Physiological and Infectious Conditions. Front. Cell Infect. Microbiol..

[58] Valdes A.M., Walter J., Segal E., Spector T.D. (2018). Role of the gut microbiota in nutrition and health. BMJ.

[59] Oliphant K., Allen-Vercoe E. (2019). Macronutrient metabolism by the human gut microbiome: Major fermentation by-products and their impact on host health. Microbiome.

[60] Ramos-Lobo A.M., Donato J. (2017). The role of leptin in health and disease. Temperature (Austin).

[61] Zeng H., Umar S., Rust B., Lazarova D., Bordonaro M. (2019). Secondary Bile Acids and Short Chain Fatty Acids in the Colon: A Focus on Colonic Microbiome, Cell Proliferation, Inflammation, and Cancer. Int. J. Mol. Sci..

[62] Schoeler M., Caesar R. (2019). Dietary lipids, gut microbiota and lipid metabolism. Rev. Endocr. Metab. Disord..

[63] Qu Y., Su C., Zhao Q., Shi A., Zhao F., Tang L., Xu D., Xiang Z., Wang Y., Wang Y. (2022). Gut Microbiota-Mediated Elevated Production of Secondary Bile Acids in Chronic Unpredictable Mild Stress. Front. Pharmacol..

[64] Hubbard T.D., Murray I.A., Perdew G.H. (2015). Indole and Tryptophan Metabolism: Endogenous and Dietary Routes to Ah Receptor Activation. Drug Metab. Dispos..

[65] Yu M., Wang Q., Ma Y., Li L., Yu K., Zhang Z., Chen G., Li X., Xiao W., Xu P. (2018). Aryl Hydrocarbon Receptor Activation Modulates Intestinal Epithelial Barrier Function by Maintaining Tight Junction Integrity. Int. J. Biol. Sci..

[66] Kwon Y.H., Wang H., Denou E., Ghia J.E., Rossi L., Fontes M.E., Bernier S.P., Shajib M.S., Banskota S., Collins S.M. (2019). Modulation of Gut Microbiota Composition by Serotonin Signaling Influences Intestinal Immune Response and Susceptibility to Colitis. Cell Mol. Gastroenterol. Hepatol..

[67] Xu M., Zhou E.Y., Shi H. (2025). Tryptophan and Its Metabolite Serotonin Impact Metabolic and Mental Disorders via the Brain–Gut–Microbiome Axis: A Focus on Sex Differences. Cells.

[68] Zheng D., Liwinski T., Elinav E. (2020). Interaction between microbiota and immunity in health and disease. Cell Res..

[69] Gubatan J., Holman D.R., Puntasecca C.J., Polevoi D., Rubin S.J., Rogalla S. (2021). Antimicrobial peptides and the gut microbiome in inflammatory bowel disease. World J. Gastroenterol..

[70] Zhao Y., Chen F., Wu W., Sun M., Bilotta A.J., Yao S., Xiao Y., Huang X., Eaves-Pyles T.D., Golovko G. (2018). GPR43 mediates microbiota metabolite SCFA regulation of antimicrobial peptide expression in intestinal epithelial cells via activation of mTOR and STAT3. Mucosal Immunol..

[71] Vaishnava S., Yamamoto M., Severson K.M., Ruhn K.A., Yu X., Koren O., Ley R., Wakeland E.K., Hooper L.V. (2011). The antibacterial lectin RegIIIgamma promotes the spatial segregation of microbiota and host in the intestine. Science.

[72] Milazzo G., Mercatelli D., Di Muzio G., Triboli L., De Rosa P., Perini G., Giorgi F.M. (2020). Histone Deacetylases (HDACs): Evolution, Specificity, Role in Transcriptional Complexes, and Pharmacological Actionability. Genes.

[73] Yang G., Chen S., Deng B., Tan C., Deng J., Zhu G., Yin Y., Ren W. (2018). Implication of G Protein-Coupled Receptor 43 in Intestinal Inflammation: A Mini-Review. Front. Immunol..

[74] Li J.H., Chen Y., Ye Z.H., Chen L.P., Xu J.X., Han J., Xie L., Xing S., Tian D.A., Seidler U. (2024). Suppression of MyD88 disturbs gut microbiota and activates the NLR pathway and hence fails to ameliorate DSS-induced colitis. Precis. Clin. Med..

[75] Price A.E., Shamardani K., Lugo K.A., Deguine J., Roberts A.W., Lee B.L., Barton G.M. (2018). A Map of Toll-like Receptor Expression in the Intestinal Epithelium Reveals Distinct Spatial, Cell Type-Specific, and Temporal Patterns. Immunity.

[76] Wang S., Charbonnier L.M., Noval Rivas M., Georgiev P., Li N., Gerber G., Bry L., Chatila T.A. (2015). MyD88 Adaptor-Dependent Microbial Sensing by Regulatory T Cells Promotes Mucosal Tolerance and Enforces Commensalism. Immunity.

[77] Manshouri S., Seif F., Kamali M., Bahar M.A., Mashayekh A., Molatefi R. (2024). The interaction of inflammasomes and gut microbiota: Novel therapeutic insights. Cell Commun. Signal..

[78] Elinav E., Strowig T., Kau A.L., Henao-Mejia J., Thaiss C.A., Booth C.J., Peaper D.R., Bertin J., Eisenbarth S.C., Gordon J.I. (2011). NLRP6 inflammasome regulates colonic microbial ecology and risk for colitis. Cell.

[79] Ouyang W., Kolls J.K., Zheng Y. (2008). The biological functions of T helper 17 cell effector cytokines in inflammation. Immunity.

[80] Raskov H., Orhan A., Christensen J.P., Gögenur I. (2021). Cytotoxic CD8^+^ T cells in cancer and cancer immunotherapy. Br. J. Cancer..

[81] Park M.H., Hong J.T. (2016). Roles of NF-κB in Cancer and Inflammatory Diseases and Their Therapeutic Approaches. Cells.

[82] Liu H., Wang J., He T., Becker S., Zhang G., Li D., Ma X. (2018). Butyrate: A Double-Edged Sword for Health?. Adv. Nutr..

[83] Wang J., Gu X., Yang J., Wei Y., Zhao Y. (2019). Gut Microbiota Dysbiosis and Increased Plasma LPS and TMAO Levels in Patients With Preeclampsia. Front. Cell Infect. Microbiol..

[84] Thye A.Y., Bah Y.R., Law J.W., Tan L.T., He Y.W., Wong S.H., Thurairajasingam S., Chan K.G., Lee L.H., Letchumanan V. (2022). Gut-Skin Axis: Unravelling the Connection between the Gut Microbiome and Psoriasis. Biomedicines.

[85] Yan D., Issa N., Afifi L., Jeon C., Chang H.W., Liao W. (2017). The Role of the Skin and Gut Microbiome in Psoriatic Disease. Curr. Dermatol. Rep..

[86] Halling M.L., Kjeldsen J., Knudsen T., Nielsen J., Hansen L.K. (2017). Patients with inflammatory bowel disease have increased risk of autoimmune and inflammatory diseases. World J. Gastroenterol..

[87] Shan Y., Lee M., Chang E.B. (2022). The Gut Microbiome and Inflammatory Bowel Diseases. Annu. Rev. Med..

[88] Sanchez-Lopez M.F., Barrero-Caicedo P.A., Olmos-Carval H.M., Torres-Medina A.F., Alzate-Granados J.P. (2025). Relationship between skin and gut microbiota dysbiosis and inflammatory skin diseases in adult patients: A systematic review. Microbe.

[89] Hrncir T. (2022). Gut Microbiota Dysbiosis: Triggers, Consequences, Diagnostic and Therapeutic Options. Microorganisms.

[90] Salem I., Ramser A., Isham N., Ghannoum M.A. (2018). The Gut Microbiome as a Major Regulator of the Gut-Skin Axis. Front. Microbiol..

[91] Wan Y., Wang F., Yuan J., Li J., Jiang D., Zhang J., Li H., Wang R., Tang J., Huang T. (2019). Effects of dietary fat on gut microbiota and faecal metabolites, and their relationship with cardiometabolic risk factors: A 6-month randomised controlled-feeding trial. Gut.

[92] Xiao W., Chen M., Peng Q., Sha K., Liu T., Xia J., Xie H., Li J., Xu S., Deng Z. (2022). Lithocholic acid promotes rosacea-like skin inflammation via G protein-coupled bile acid receptor 1. Biochim. Biophys. Acta Mol. Basis Dis..

[93] Gillard J., Roumain M., Picalausa C., Thibaut M.M., Clerbaux L.A., Tailleux A., Staels B., Muccioli G.G., Bindels L.B., Leclercq I.A. (2024). A gut microbiota-independent mechanism shapes the bile acid pool in mice with MASH. JHEP Rep..

[94] Lavoie H., Gagnon J., Therrien M. (2020). ERK signalling: A master regulator of cell behaviour, life and fate. Nat. Rev. Mol. Cell Biol..

[95] Ye D., He J., He X. (2024). The role of bile acid receptor TGR5 in regulating inflammatory signalling. Scand. J. Immunol..

[96] Stojanov S., Berlec A., Štrukelj B. (2020). The Influence of Probiotics on the Firmicutes/Bacteroidetes Ratio in the Treatment of Obesity and Inflammatory Bowel disease. Microorganisms.

[97] Subramaniam S., Fletcher C. (2018). Trimethylamine N-oxide: Breathe new life. Br. J. Pharmacol..

[98] Gatarek P., Kaluzna-Czaplinska J. (2021). Trimethylamine N-oxide (TMAO) in human health. EXCLI J..

[99] Blevins H.M., Xu Y., Biby S., Zhang S. (2022). The NLRP3 Inflammasome Pathway: A Review of Mechanisms and Inhibitors for the Treatment of Inflammatory Diseases. Front. Aging Neurosci..

[100] Yang S., Li X., Yang F., Zhao R., Pan X., Liang J., Tian L., Li X., Liu L., Xing Y. (2019). Gut Microbiota-Dependent Marker TMAO in Promoting Cardiovascular Disease: Inflammation Mechanism, Clinical Prognostic, and Potential as a Therapeutic Target. Front. Pharmacol..

[101] Kim R.B., Morse B.L., Djurdjev O., Tang M., Muirhead N., Barrett B., Holmes D.T., Madore F., Clase C.M., Rigatto C. (2016). Advanced chronic kidney disease populations have elevated trimethylamine N-oxide levels associated with increased cardiovascular events. Kidney Int..

[102] Al-Rubaye H., Perfetti G., Kaski J.C. (2019). The Role of Microbiota in Cardiovascular Risk: Focus on Trimethylamine Oxide. Curr. Probl. Cardiol..

[103] Barrea L., Muscogiuri G., Pugliese G., de Alteriis G., Maisto M., Donnarumma M., Tenore G.C., Colao A., Fabbrocini G., Savastano S. (2021). Association of Trimethylamine N-Oxide (TMAO) with the Clinical Severity of Hidradenitis Suppurativa (Acne Inversa). Nutrients.

[104] Stepaniuk A., Baran A., Flisiak I. (2023). Kynurenine Pathway in Psoriasis-a Promising Link?. Dermatol. Ther..

[105] Miri S., Yeo J., Abubaker S., Hammami R. (2023). Neuromicrobiology, an emerging neurometabolic facet of the gut microbiome?. Front. Microbiol..

[106] Sittipo P., Choi J., Lee S., Lee Y.K. (2022). The function of gut microbiota in immune-related neurological disorders: A review. J. Neuroinflamm..

[107] Sandoval-Talamantes A.K., Gómez-González B.A., Uriarte-Mayorga D.F., Martínez-Guzman M.A., Wheber-Hidalgo K.A., Alvarado-Navarro A. (2020). Neurotransmitters, neuropeptides and their receptors interact with immune response in healthy and psoriatic skin. Neuropeptides.

[108] Marek-Jozefowicz L., Czajkowski R., Borkowska A., Nedoszytko B., Żmijewski M.A., Cubała W.J., Slominski A.T. (2022). The Brain-Skin Axis in Psoriasis-Psychological, Psychiatric, Hormonal, and Dermatological Aspects. Int. J. Mol. Sci..

[109] O’Neill C.A., Monteleone G., McLaughlin J.T., Paus R. (2016). The gut-skin axis in health and disease: A paradigm with therapeutic implications. Bioessays.

[110] Miyazaki K., Masuoka N., Kano M., Iizuka R. (2014). Bifidobacterium fermented milk and galacto-oligosaccharides lead to improved skin health by decreasing phenols production by gut microbiota. Benef. Microbes..

[111] Buret A.G., Allain T., Motta J.P., Wallace J.L. (2022). Effects of Hydrogen Sulfide on the Microbiome: From Toxicity to Therapy. Antioxid. Redox Signal..

[112] Teigen L., Mathai P.P., Lopez S., Matson M., Elkin B., Kozysa D., Kabage A.J., Hamilton M., Vaughn B.P., Sadowsky M.J. (2022). Differential hydrogen sulfide production by a human cohort in response to animal- and plant-based diet interventions. Clin. Nutr..

[113] Gorini F., Del Turco S., Sabatino L., Gaggini M., Vassalle C. (2021). H_2_S as a Bridge Linking Inflammation, Oxidative Stress and Endothelial Biology: A Possible Defense in the Fight against SARS-CoV-2 Infection?. Biomedicines.

[114] Xiao Q., Xiong L., Tang J., Li L., Li L. (2021). Hydrogen Sulfide in Skin Diseases: A Novel Mediator and Therapeutic Target. Oxid. Med. Cell Longev..

[115] Coavoy-Sánchez S.A., Costa S.K.P., Muscará M.N. (2020). Hydrogen sulfide and dermatological diseases. Br. J. Pharmacol..

[116] Xu Q., Ni J.J., Han B.X., Yan S.S., Wei X.T., Feng G.J., Zhang H., Zhang L., Li B., Pei Y.F. (2022). Causal Relationship Between Gut Microbiota and Autoimmune Diseases: A Two-Sample Mendelian Randomization Study. Front. Immunol..

[117] Wang Y., Xia X., Zhou X., Zhan T., Dai Q., Zhang Y., Zhang W., Shu Y., Li W., Xu H. (2023). Association of gut microbiome and metabolites with onset and treatment response of patients with pemphigus vulgaris. Front. Immunol..

[118] Stirnadel-Farrant H.A., Xu X., Kwiatek J., Jain P., Meyers J., Candrilli S., Mines D., Datto C.J. (2023). Characteristics, treatment patterns, health care resource utilization and costs in patients with bullous pemphigoid: A retrospective analysis of US health insurance claims data. JAAD Int..

[119] Hsu D., Brieva J., Silverberg J.I. (2016). Costs of Care for Hospitalization for Pemphigus in the United States. JAMA Dermatol..

[120] Walko G., Castañón M.J., Wiche G. (2015). Molecular architecture and function of the hemidesmosome. Cell Tissue Res..

[121] Genovese G., Di Zenzo G., Cozzani E., Berti E., Cugno M., Marzano A.V. (2019). New Insights Into the Pathogenesis of Bullous Pemphigoid: 2019 Update. Front. Immunol..

[122] Hesari R., Thibaut D., Schur N., Thoutireddy S., Witcher R., Julian E. (2023). Bullous Pemphigoid and Human Leukocyte Antigen (HLA)-DQA1: A Systematic Review. Cureus.

[123] Huang R., Hu L., Jiang F. (2023). Study of cytokine-induced immunity in bullous pemphigoid: Recent developments. Ann. Med..

[124] Gupta A., Dhakan D.B., Maji A., Saxena R., PK V.P., Mahajan S., Pulikkan J., Kurian J., Gomez A.M., Scaria J. (2019). Association of *Flavonifractor plautii*, a Flavonoid-Degrading Bacterium, with the Gut Microbiome of Colorectal Cancer Patients in India. mSystems.

[125] Ogita T., Yamamoto Y., Mikami A., Shigemori S., Sato T., Shimosato T. (2020). Oral Administration of *Flavonifractor plautii* Strongly Suppresses Th2 Immune Responses in Mice. Front. Immunol..

[126] Mikami A., Ogita T., Namai F., Shigemori S., Sato T., Shimosato T. (2020). Oral administration of *Flavonifractor plautii* attenuates inflammatory responses in obese adipose tissue. Mol. Biol. Rep..

[127] Mikami A., Ogita T., Namai F., Shigemori S., Sato T., Shimosato T. (2021). Oral Administration of *Flavonifractor plautii*, a Bacteria Increased With Green Tea Consumption, Promotes Recovery From Acute Colitis in Mice via Suppression of IL-17. Front. Nutr..

[128] Qiu X., Zhang M., Yang X., Hong N., Yu C. (2013). *Faecalibacterium prausnitzii* upregulates regulatory T cells and anti-inflammatory cytokines in treating TNBS-induced colitis. J. Crohns Colitis..

[129] Ferreira-Halder C.V., Faria A.V.S., Andrade S.S. (2017). Action and function of *Faecalibacterium prausnitzii* in health and disease. Best. Pract. Res. Clin. Gastroenterol..

[130] Prosberg M., Bendtsen F., Vind I., Petersen A.M., Gluud L.L. (2016). The association between the gut microbiota and the inflammatory bowel disease activity: A systematic review and meta-analysis. Scand. J. Gastroenterol..

[131] El Atty K.A., Nouh H., Abdelsalam S., Ellakany A., Abdaalah H., Header D. (2024). Study of *Fecalibacteria prausntzii* in Egyptian patients with inflammatory bowel disease. Prz. Gastroenterol..

[132] Dikeocha I.J., Al-Kabsi A.M., Chiu H.T., Alshawsh M.A. (2022). *Faecalibacterium prausnitzii* Ameliorates Colorectal Tumorigenesis and Suppresses Proliferation of HCT116 Colorectal Cancer Cells. Biomedicines.

[133] Ganesan K., Chung S.K., Vanamala J., Xu B. (2018). Causal Relationship between Diet-Induced Gut Microbiota Changes and Diabetes: A Novel Strategy to Transplant *Faecalibacterium prausnitzii* in Preventing Diabetes. Int. J. Mol. Sci..

[134] Quillin S.J., Tran P., Prindle A. (2021). Potential Roles for Gamma-Aminobutyric Acid Signaling in Bacterial Communities. Bioelectricity..

[135] Ham S., Bhatia S.K., Gurav R., Choi Y.K., Jeon J.M., Yoon J.J., Choi K.Y., Ahn J., Kim H.T., Yang Y.H. (2022). Gamma aminobutyric acid (GABA) production in *Escherichia coli* with pyridoxal kinase (pdxY) based regeneration system. Enzyme Microb. Technol..

[136] Grosheva I., Zheng D., Levy M., Polansky O., Lichtenstein A., Golani O., Dori-Bachash M., Moresi C., Shapiro H., Del Mare-Roumani S. (2020). High-Throughput Screen Identifies Host and Microbiota Regulators of Intestinal Barrier Function. Gastroenterology.

[137] Chang H.W., Yan D., Singh R., Bui A., Lee K., Truong A., Milush J.M., Somsouk M., Liao W. (2022). Multiomic Analysis of the Gut Microbiome in Psoriasis Reveals Distinct Host—Microbe Associations. JID Innov..

[138] Miller-Fleming L., Olin-Sandoval V., Campbell K., Ralser M. (2015). Remaining Mysteries of Molecular Biology: The Role of Polyamines in the Cell. J. Mol. Biol..

[139] Tofalo R., Cocchi S., Suzzi G. (2019). Polyamines and Gut Microbiota. Front. Nutr..

[140] Thirion F., Guilly S., Fromentin S., Plaza Oñate F., Alvarez A.S., Le Chatelier E., Pons N., Levenez F., Quinquis B., Ehrlich S. (2022). Changes in Gut Microbiota of Patients with Atopic Dermatitis During Balneotherapy. Clin. Cosmet. Investig. Dermatol..

[141] Andermann T., Antonelli A., Barrett R.L., Silvestro D. (2022). Estimating Alpha, Beta, and Gamma Diversity Through Deep Learning. Front. Plant Sci..

[142] Sinha S.R., Haileselassie Y., Nguyen L.P., Tropini C., Wang M., Becker L.S., Sim D., Jarr K., Spear E.T., Singh G. (2020). Dysbiosis-Induced Secondary Bile Acid Deficiency Promotes Intestinal Inflammation. Cell Host Microbe.

[143] Lobionda S., Sittipo P., Kwon H.Y., Lee Y.K. (2019). The Role of Gut Microbiota in Intestinal Inflammation with Respect to Diet and Extrinsic Stressors. Microorganisms.

[144] West C.E., Rydén P., Lundin D., Engstrand L., Tulic M.K., Prescott S.L. (2015). Gut microbiome and innate immune response patterns in IgE-associated eczema. Clin. Exp. Allergy..

[145] Birzele L.T., Depner M., Ege M.J., Engel M., Kublik S., Bernau C., Loss G.J., Genuneit J., Horak E., Schloter M. (2017). Environmental and mucosal microbiota and their role in childhood asthma. Allergy.

[146] Manasson J., Shen N., Garcia Ferrer H.R., Ubeda C., Iraheta I., Heguy A., Von Feldt J.M., Espinoza L.R., Garcia Kutzbach A., Segal L.N. (2018). Gut Microbiota Perturbations in Reactive Arthritis and Postinfectious Spondyloarthritis. Arthritis Rheumatol..

[147] Ćesić D., Lugović Mihić L., Ozretić P., Lojkić I., Buljan M., Šitum M., Zovak M., Vidović D., Mijić A., Galić N. (2023). Association of Gut *Lachnospiraceae* and Chronic Spontaneous Urticaria. Life.

[148] Zaplana T., Miele S., Tolonen A.C. (2024). Lachnospiraceae are emerging industrial biocatalysts and biotherapeutics. Front. Bioeng. Biotechnol..

[149] Lu J., Zhang P., Hu R., Qi S., Zhao Y., Miao Y., Han Y., Zhou L., Yang Q. (2021). Gut microbiota characterization in Chinese patients with alopecia areata. J. Dermatol. Sci..

[150] Lam S.Y., Radjabzadeh D., Eppinga H., Nossent Y.R.A., van der Zee H.H., Kraaij R., Konstantinov S.R., Fuhler G.M., Prens E.P., Thio H.B. (2021). A microbiome study to explore the gut-skin axis in hidradenitis suppurativa. J. Dermatol. Sci..

[151] Wang Y., Ames N.P., Tun H.M., Tosh S.M., Jones P.J., Khafipour E. (2016). High Molecular Weight Barley β-Glucan Alters Gut Microbiota Toward Reduced Cardiovascular Disease Risk. Front. Microbiol..

[152] Precup G., Vodnar D.C. (2019). Gut *Prevotella* as a possible biomarker of diet and its eubiotic versus dysbiotic roles: A comprehensive literature review. Br. J. Nutr..

[153] Huang Y., Tang J., Cai Z., Zhou K., Chang L., Bai Y., Ma Y. (2020). *Prevotella* Induces the Production of Th17 Cells in the Colon of Mice. J. Immunol. Res..

[154] Scher J.U., Sczesnak A., Longman R.S., Segata N., Ubeda C., Bielski C., Rostron T., Cerundolo V., Pamer E.G., Abramson S.B. (2013). Expansion of intestinal *Prevotella copri* correlates with enhanced susceptibility to arthritis. Elife.

[155] Dillon S.M., Lee E.J., Kotter C.V., Austin G.L., Dong Z., Hecht D.K., Gianella S., Siewe B., Smith D.M., Landay A.L. (2014). An altered intestinal mucosal microbiome in HIV-1 infection is associated with mucosal and systemic immune activation and endotoxemia. Mucosal Immunol..

[156] Pedersen H.K., Gudmundsdottir V., Nielsen H.B., Hyotylainen T., Nielsen T., Jensen B.A., Forslund K., Hildebrand F., Prifti E., Falony G. (2016). Human gut microbes impact host serum metabolome and insulin sensitivity. Nature.

[157] Wen C., Zheng Z., Shao T., Liu L., Xie Z., Le Chatelier E., He Z., Zhong W., Fan Y., Zhang L. (2017). Quantitative metagenomics reveals unique gut microbiome biomarkers in ankylosing spondylitis. Genome Biol..

[158] De Vadder F., Kovatcheva-Datchary P., Zitoun C., Duchampt A., Bäckhed F., Mithieux G. (2016). Microbiota-Produced Succinate Improves Glucose Homeostasis via Intestinal Gluconeogenesis. Cell Metab..

[159] Kovatcheva-Datchary P., Nilsson A., Akrami R., Lee Y.S., De Vadder F., Arora T., Hallen A., Martens E., Björck I., Bäckhed F. (2015). Dietary Fiber-Induced Improvement in Glucose Metabolism Is Associated with Increased Abundance of *Prevotella*. Cell Metab..

[160] De Filippis F., Pasolli E., Tett A., Tarallo S., Naccarati A., De Angelis M., Neviani E., Cocolin L., Gobbetti M., Segata N. (2019). Distinct Genetic and Functional Traits of Human Intestinal *Prevotella* copri Strains Are Associated with Different Habitual Diets. Cell Host Microbe.

[161] Tett A., Huang K.D., Asnicar F., Fehlner-Peach H., Pasolli E., Karcher N., Armanini F., Manghi P., Bonham K., Zolfo M. (2019). The *Prevotella copri* Complex Comprises Four Distinct Clades Underrepresented in Westernized Populations. Cell Host Microbe.

[162] Zhang S.M., Huang S.L. (2023). The Commensal Anaerobe *Veillonella dispar* Reprograms Its Lactate Metabolism and Short-Chain Fatty Acid Production during the Stationary Phase. Microbiol. Spectr..

[163] Fultz R., Ticer T., Ihekweazu F.D., Horvath T.D., Haidacher S.J., Hoch K.M., Bajaj M., Spinler J.K., Haag A.M., Buffington S.A. (2021). Unraveling the Metabolic Requirements of the Gut Commensal *Bacteroides ovatus*. Front. Microbiol..

[164] Han Z., Fan Y., Wu Q., Guo F., Li S., Hu X., Zuo Y.G. (2024). Comparison of gut microbiota dysbiosis between pemphigus vulgaris and bullous pemphigoid. Int. Immunopharmacol..

[165] Mazgaeen L., Gurung P. (2020). Recent Advances in Lipopolysaccharide Recognition Systems. Int. J. Mol. Sci..

[166] You S., Ouyang J., Wu Q., Zhang Y., Gao J., Luo X., Wang Y., Wu Y., Jiang F. (2024). Comparison of serum cytokines and chemokines levels and clinical significance in patients with pemphigus vulgaris-A retrospective study. Exp. Dermatol..

[167] Timoteo R.P., da Silva M.V., Miguel C.B., Silva D.A., Catarino J.D., Rodrigues Junior V., Sales-Campos H., Freire Oliveira C.J. (2017). Th1/Th17-Related Cytokines and Chemokines and Their Implications in the Pathogenesis of Pemphigus Vulgaris. Mediators Inflamm..

[168] Abdugheni R., Wang W.Z., Wang Y.J., Du M.X., Liu F.L., Zhou N., Jiang C.Y., Wang C.Y., Wu L., Ma J. (2022). Metabolite profiling of human-originated Lachnospiraceae at the strain level. Imeta.

[169] Gu B.H., Choi J.P., Park T., Kim A.S., Jung H.Y., Choi D.Y., Lee S.J., Chang Y.S., Kim M., Park H.K. (2023). Adult asthma with symptomatic eosinophilic inflammation is accompanied by alteration in gut microbiome. Allergy.

[170] Notting F., Pirovano W., Sybesma W., Kort R. (2023). The butyrate-producing and spore-forming bacterial genus *Coprococcus* as a potential biomarker for neurological disorders. Gut Microbiome.

[171] Coello K., Hansen T.H., Sørensen N., Ottesen N.M., Miskowiak K.W., Pedersen O., Kessing L.V., Vinberg M. (2021). Affective disorders impact prevalence of *Flavonifractor* and abundance of Christensenellaceae in gut microbiota. Prog. Neuropsychopharmacol. Biol. Psychiatry.

[172] Yang Y., Du L., Shi D., Kong C., Liu J., Liu G., Li X., Ma Y. (2021). Dysbiosis of human gut microbiome in young-onset colorectal cancer. Nat. Commun..

[173] Guo Z., Yiu N., Hu Z., Zhou W., Long X., Yang M., Liao J., Zhang G., Lu Q., Zhao M. (2023). Alterations of fecal microbiome and metabolome in pemphigus patients. J. Autoimmun..

[174] Daniel B.S., Hertl M., Werth V.P., Eming R., Murrell D.F. (2012). Severity score indexes for blistering diseases. Clin. Dermatol..

[175] Calzada E., Onguka O., Claypool S.M. (2016). Phosphatidylethanolamine Metabolism in Health and Disease. Int. Rev. Cell Mol. Biol..

[176] Anand P.K. (2020). Lipids, inflammasomes, metabolism, and disease. Immunol. Rev..

[177] Mu Q., Kirby J., Reilly C.M., Luo X.M. (2017). Leaky Gut As a Danger Signal for Autoimmune Diseases. Front. Immunol..

[178] Singh V., Yeoh B.S., Xiao X., Kumar M., Bachman M., Borregaard N., Joe B., Vijay-Kumar M. (2015). Interplay between enterobactin, myeloperoxidase and lipocalin 2 regulates E. coli survival in the inflamed gut. Nat. Commun..

[179] Houot L., Chang S., Pickering B.S., Absalon C., Watnick P.I. (2010). The phosphoenolpyruvate phosphotransferase system regulates *Vibrio cholerae* biofilm formation through multiple independent pathways. J. Bacteriol..

[180] Arrieta M.C., Stiemsma L.T., Dimitriu P.A., Thorson L., Russell S., Yurist-Doutsch S., Kuzeljevic B., Gold M.J., Britton H.M., Lefebvre D.L. (2015). Early infancy microbial and metabolic alterations affect risk of childhood asthma. Sci. Transl. Med..

[181] Hasegawa K., Linnemann R.W., Mansbach J.M., Ajami N.J., Espinola J.A., Petrosino J.F., Piedra P.A., Stevenson M.D., Sullivan A.F., Thompson A.D. (2016). The Fecal Microbiota Profile and Bronchiolitis in Infants. Pediatrics.

[182] Larsen J.M. (2017). The immune response to *Prevotella* bacteria in chronic inflammatory disease. Immunology.

[183] Sharma G., Garg N., Hasan S., Shirodkar S. (2022). *Prevotella*: An insight into its characteristics and associated virulence factors. Microb. Pathog..

[184] Konuma T., Kohara C., Watanabe E., Takahashi S., Ozawa G., Inomata K., Suzuki K., Mizukami M., Nagai E., Okabe M. (2020). Impact of Intestinal Microbiota on Reconstitution of Circulating Monocyte, Dendritic Cell, and Natural Killer Cell Subsets in Adults Undergoing Single-Unit Cord Blood Transplantation. Biol. Blood Marrow Transplant..

[185] Xiao Y., Shi Y., Ni Y., Ni M., Yang Y., Zhang X. (2024). Gestational diabetes-combined excess weight gain exacerbates gut microbiota dysbiosis in newborns, associated with reduced abundance of *Clostridium*, *Coriobacteriaceae*, and *Collinsella*. Front. Cell Infect. Microbiol..

[186] Houtman T.A., Eckermann H.A., Smidt H., de Weerth C. (2022). Gut microbiota and BMI throughout childhood: The role of firmicutes, bacteroidetes, and short-chain fatty acid producers. Sci. Rep..

[187] Chang S.H., Choi Y. (2023). Gut dysbiosis in autoimmune diseases: Association with mortality. Front. Cell Infect. Microbiol..

[188] Litvak Y., Byndloss M.X., Tsolis R.M., Bäumler A.J. (2017). Dysbiotic Proteobacteria expansion: A microbial signature of epithelial dysfunction. Curr. Opin. Microbiol..

[189] Chen H., Ou R., Tang N., Su W., Yang R., Yu X., Zhang G., Jiao J., Zhou X. (2023). Alternation of the gut microbiota in irritable bowel syndrome: An integrated analysis based on multicenter amplicon sequencing data. J. Transl. Med..

[190] Xu Z., Jiang W., Huang W., Lin Y., Chan F.K.L., Ng S.C. (2022). Gut microbiota in patients with obesity and metabolic disorders—A systematic review. Genes Nutr..

[191] Guo X., Huang C., Xu J., Xu H., Liu L., Zhao H., Wang J., Huang W., Peng W., Chen Y. (2022). Gut Microbiota Is a Potential Biomarker in Inflammatory Bowel Disease. Front. Nutr..

[192] Xu Y., Zhao J., Ma Y., Liu J., Cui Y., Yuan Y., Xiang C., Ma D., Liu H. (2023). The microbiome types of colorectal tissue are potentially associated with the prognosis of patients with colorectal cancer. Front. Microbiol..

[193] Lu M., Xuan S., Wang Z. (2019). Oral microbiota: A new view of body health. Food Sci Hum Well..

[194] Baker J.L., Mark Welch J.L., Kauffman K.M., McLean J.S., He X. (2024). The oral microbiome: Diversity, biogeography and human health. Nat. Rev. Microbiol..

[195] Fan W., Lei N., Zheng Y., Liu J., Cao X., Su T., Su Z., Lu Y. (2024). Oral microbiota diversity in moderate to severe plaque psoriasis, nail psoriasis and psoriatic arthritis. Sci. Rep..

[196] Mosaddad S.A., Mahootchi P., Safari S., Rahimi H., Aghili S.S. (2023). Interactions between systemic diseases and oral microbiota shifts in the aging community: A narrative review. J. Basic. Microbiol..

[197] Macklis P., Adams K., Kaffenberger J., Kumar P., Krispinsky A., Kaffenberger B. (2020). The Association Between Oral Health and Skin Disease. J. Clin. Aesthet. Dermatol..

[198] Qiao P., Shi Q., Zhang R., E L., Wang P., Wang J., Liu H. (2019). Psoriasis Patients Suffer From Worse Periodontal Status-A Meta-Analysis. Front. Med..

[199] Murata T., Yamaga T., Iida T., Miyazaki H., Yaegaki K. (2002). Classification and examination of halitosis. Int Dent J..

[200] Zorba M., Melidou A., Patsatsi A., Poulopoulos A., Gioula G., Kolokotronis A., Minti F. (2021). The role of oral microbiome in pemphigus vulgaris. Arch. Microbiol..

[201] Subadra K., S S., Warrier S.A. (2021). Oral Pemphigus Vulgaris. Cureus.

[202] Murphy E.C., Frick I.M. (2013). Gram-positive anaerobic cocci–commensals and opportunistic pathogens. FEMS Microbiol. Rev..

[203] Coker O.O., Dai Z., Nie Y., Zhao G., Cao L., Nakatsu G., Wu W.K., Wong S.H., Chen Z., Sung J.J.Y. (2018). Mucosal microbiome dysbiosis in gastric carcinogenesis. Gut.

[204] Flemer B., Warren R.D., Barrett M.P., Cisek K., Das A., Jeffery I.B., Hurley E., O’Riordain M., Shanahan F., O’Toole P.W. (2018). The oral microbiota in colorectal cancer is distinctive and predictive. Gut.

[205] Higashi D.L., Krieger M.C., Qin H., Zou Z., Palmer E.A., Kreth J., Merritt J. (2023). Who is in the driver’s seat? *Parvimonas micra*: An understudied pathobiont at the crossroads of dysbiotic disease and cancer. Environ. Microbiol. Rep..

[206] Signat B., Roques C., Poulet P., Duffaut D. (2011). *Fusobacterium nucleatum* in periodontal health and disease. Curr. Issues Mol. Biol..

[207] Komiya Y., Shimomura Y., Higurashi T., Sugi Y., Arimoto J., Umezawa S., Uchiyama S., Matsumoto M., Nakajima A. (2019). Patients with colorectal cancer have identical strains of *Fusobacterium nucleatum* in their colorectal cancer and oral cavity. Gut.

[208] Pignatelli P., Nuccio F., Piattelli A., Curia M.C. (2023). The Role of *Fusobacterium nucleatum* in Oral and Colorectal Carcinogenesis. Microorganisms.

[209] Chiscuzzu F., Crescio C., Varrucciu S., Rizzo D., Sali M., Delogu G., Bussu F. (2025). Current Evidence on the Relation Between Microbiota and Oral Cancer-The Role of *Fusobacterium nucleatum*-A Narrative Review. Cancers.

[210] Begić G., Badovinac I.J., Karleuša L., Kralik K., Cvijanovic Peloza O., Kuiš D., Gobin I. (2023). *Streptococcus salivarius* as an Important Factor in Dental Biofilm Homeostasis: Influence on *Streptococcus mutans* and *Aggregatibacter actinomycetemcomitans* in Mixed Biofilm. Int. J. Mol. Sci..

[211] Yang Y., Qu L., Mijakovic I., Wei Y. (2022). Advances in the human skin microbiota and its roles in cutaneous diseases. Microb. Cell Fact..

[212] Ito Y., Amagai M. (2023). Dissecting skin microbiota and microenvironment for the development of therapeutic strategies. Curr. Opin. Microbiol..

[213] Belkaid Y., Segre J.A. (2014). Dialogue between skin microbiota and immunity. Science.

[214] Smythe P., Wilkinson H.N. (2023). The Skin Microbiome: Current Landscape and Future Opportunities. Int. J. Mol. Sci..

[215] Yamazaki Y., Nakamura Y., Núñez G. (2017). Role of the microbiota in skin immunity and atopic dermatitis. Allergol. Int..

[216] Claudel J.P., Auffret N., Leccia M.T., Poli F., Corvec S., Dréno B. (2019). *Staphylococcus epidermidis*: A Potential New Player in the Physiopathology of Acne?. Dermatology.

[217] Chang H.W., Yan D., Singh R., Liu J., Lu X., Ucmak D., Lee K., Afifi L., Fadrosh D., Leech J. (2018). Alteration of the cutaneous microbiome in psoriasis and potential role in Th17 polarization. Microbiome.

[218] Celoria V., Rosset F., Pala V., Dapavo P., Ribero S., Quaglino P., Mastorino L. (2023). The Skin Microbiome and Its Role in Psoriasis: A Review. Psoriasis.

[219] Iwamoto K., Moriwaki M., Miyake R., Hide M. (2019). *Staphylococcus aureus* in atopic dermatitis: Strain-specific cell wall proteins and skin immunity. Allergol. Int..

[220] Rainer B.M., Thompson K.G., Antonescu C., Florea L., Mongodin E.F., Bui J., Fischer A.H., Pasieka H.B., Garza L.A., Kang S. (2020). Characterization and Analysis of the Skin Microbiota in Rosacea: A Case-Control Study. Am. J. Clin. Dermatol..

[221] Tutka K., Żychowska M., Reich A. (2020). Diversity and Composition of the Skin, Blood and Gut Microbiome in Rosacea-A Systematic Review of the Literature. Microorganisms.

[222] Woo Y.R., Lee S.H., Cho S.H., Lee J.D., Kim H.S. (2020). Characterization and Analysis of the Skin Microbiota in Rosacea: Impact of Systemic Antibiotics. J. Clin. Med..

[223] Ferček I., Lugović-Mihić L., Tambić-Andrašević A., Ćesić D., Grginić A.G., Bešlić I., Mravak-Stipetić M., Mihatov-Štefanović I., Buntić A.M., Čivljak R. (2021). Features of the Skin Microbiota in Common Inflammatory Skin Diseases. Life.

[224] Flowers L., Grice E.A. (2020). The Skin Microbiota: Balancing Risk and Reward. Cell Host Microbe.

[225] Hong J., Buddenkotte J., Berger T.G., Steinhoff M. (2011). Management of itch in atopic dermatitis. Semin. Cutan. Med. Surg..

[226] Billeci L., Tonacci A., Tartarisco G., Ruta L., Pioggia G., Gangemi S. (2015). Association Between Atopic Dermatitis and Autism Spectrum Disorders: A Systematic Review. Am. J. Clin. Dermatol..

[227] Hashimoto T., Ohzono A., Teye K., Numata S., Hiroyasu S., Tsuruta D., Hachiya T., Kuroda K., Hashiguchi M., Kawakami T. (2017). Detection of IgE autoantibodies to BP180 and BP230 and their relationship to clinical features in bullous pemphigoid. Br. J. Dermatol..

[228] Murdaca G., Greco M., Tonacci A., Negrini S., Borro M., Puppo F., Gangemi S. (2019). IL-33/IL-31 Axis in Immune-Mediated and Allergic Diseases. Int. J. Mol. Sci..

[229] Chen Y.E., Fischbach M.A., Belkaid Y. (2018). Skin microbiota-host interactions. Nature.

[230] Messingham K.N., Cahill M.P., Kilgore S.H., Munjal A., Schlievert P.M., Fairley J.A. (2022). TSST-1^+^
*Staphylococcus aureus* in Bullous Pemphigoid. J. Investig. Dermatol..

[231] Conti F., Ceccarelli F., Iaiani G., Perricone C., Giordano A., Amori L., Miranda F., Massaro L., Pacucci V.A., Truglia S. (2016). Association between *Staphylococcus aureus* nasal carriage and disease phenotype in patients affected by systemic lupus erythematosus. Arthritis Res. Ther..

[232] Gimza B.D., Cassat J.E. (2021). Mechanisms of Antibiotic Failure During *Staphylococcus aureus* Osteomyelitis. Front. Immunol..

[233] Gobao V.C., Alfishawy M., Smith C., Byers K.E., Yassin M., Urish K.L., Shah N.B. (2020). Risk Factors, Screening, and Treatment Challenges in *Staphylococcus aureus* Native Septic Arthritis. Open Forum Infect. Dis..

[234] Powers M.E., Bubeck Wardenburg J. (2014). Igniting the fire: Staphylococcus aureus virulence factors in the pathogenesis of sepsis. PLoS Pathog..

[235] van der Kooi-Pol M.M., Duipmans J.C., Jonkman M.F., van Dijl J.M. (2014). Host-pathogen interactions in epidermolysis bullosa patients colonized with *Staphylococcus aureus*. Int. J. Med. Microbiol..

[236] Belheouane M., Hermes B.M., Van Beek N., Benoit S., Bernard P., Drenovska K., Gerdes S., Gläser R., Goebeler M., Günther C. (2023). Characterization of the skin microbiota in bullous pemphigoid patients and controls reveals novel microbial indicators of disease. J. Adv. Res..

[237] Srinivas G., Möller S., Wang J., Künzel S., Zillikens D., Baines J.F., Ibrahim S.M. (2013). Genome-wide mapping of gene-microbiota interactions in susceptibility to autoimmune skin blistering. Nat. Commun..

[238] Yerushalmi M., Elalouf O., Anderson M., Chandran V. (2019). The skin microbiome in psoriatic disease: A systematic review and critical appraisal. J. Transl. Autoimmun..

[239] Rauer L., Reiger M., Bhattacharyya M., Brunner P.M., Krueger J.G., Guttman-Yassky E., Traidl-Hoffmann C., Neumann A.U. (2023). Skin microbiome and its association with host cofactors in determining atopic dermatitis severity. J. Eur. Acad. Dermatol. Venereol..

[240] Shu M., Wang Y., Yu J., Kuo S., Coda A., Jiang Y., Gallo R.L., Huang C.M. (2013). Fermentation of *Propionibacterium acnes*, a commensal bacterium in the human skin microbiome, as skin probiotics against methicillin-resistant *Staphylococcus aureus*. PLoS ONE.

[241] Nakamura K., O’Neill A.M., Williams M.R., Cau L., Nakatsuji T., Horswill A.R., Gallo R.L. (2020). Short chain fatty acids produced by *Cutibacterium acnes* inhibit biofilm formation by Staphylococcus epidermidis. Sci. Rep..

[242] Mayslich C., Grange P.A., Dupin N. (2021). *Cutibacterium acnes* as an Opportunistic Pathogen: An Update of Its Virulence-Associated Factors. Microorganisms.

